# Formative research for a pre-operative psychosocial screening program for cardiac surgical patients: The EMBRACE study, a mixed methods knowledge to action protocol

**DOI:** 10.1371/journal.pone.0322592

**Published:** 2025-12-30

**Authors:** Susan E. Smith, Marlien Varnfield, David J. Kavanagh, Tricia Rolls, Usha Gurunathan, Bo Janoschka, Karen Hay, Rishendran Naidoo, Jed Duff, Esben Strodl

**Affiliations:** 1 Cardiothoracic Surgery Service, The Prince Charles Hospital, Chermside, Queensland, Australia; 2 School of Psychology and Counselling, Queensland University of Technology, Kelvin Grove, Queensland, Australia; 3 Australian e-Health Research Centre, Commonwealth Scientific and Industrial Research Organisation, Herston, Queensland, Australia; 4 Psychology Department, The Prince Charles Hospital, Chermside, Queensland, Australia; 5 Department of Anaesthesia and Perfusion Services, The Prince Charles Hospital, Chermside, Queensland, Australia; 6 Faculty of Medicine, The University of Queensland, Brisbane, Australia; 7 Cardiology Clinical Research Centre, The Prince Charles Hospital, Chermside, Queensland, Australia; 8 QIMR Berghofer Medical Research Institute, Herston, Queensland, Australia; 9 Centre for Healthcare Transformation, Royal Brisbane and Women’s Hospital, Herston, Queensland, Australia; 10 School of Nursing, Queensland University of Technology, Kelvin Grove, Queensland, Australia; Sengkang General Hospital, SINGAPORE

## Abstract

**Background:**

Psychological comorbidities are common in cardiac surgery patients, however much research on their prevalence, correlates and effects remains subject to methodological inconsistencies, with screening and interventions to address the problem not being systematically applied. More information about patient preferences for support and formative contextual knowledge is needed to improve screening program design, uptake, and benefits.

**Aims:**

This study aims to estimate the prevalence and relationship of psychosocial comorbidity with cardiac surgery post-operative health outcomes and explore patients’ support interests and preferences in the public hospital acute surgical setting. These findings will generate contextualised knowledge for subsequent development and implementation of a psychosocial screening and support program.

**Methods:**

A sample of 260 patients will be screened using a pragmatic informatics platform of pre-operatively self-reported psychometric instruments including for depression, anxiety, PTSD, perceived stress, and personality traits. Post-operative outcomes and medical covariates will be linked from routinely collected clinical data as well as post-operative psychometric surveys. Prevalence of exposures of interest will be ascertained, and multivariable regression will assess associations with the primary outcome of Days Alive and Out of Hospital to 30 days (DAOH), controlling for patient-level covariates. Secondary outcomes will include measures of post-operative morbidity, Quality of Life and resource utilisation to 1 year of follow-up. Simultaneously, mixed methods will be used to elucidate patient interests and preferences for available support options including online eMental Health resources in blended care, via a post-operative preferences survey and a nested subsample of semi-structured interviews.

**Conclusion:**

Inconsistent evidence on screening program implementation and patient benefit necessitates a re-evaluation of locally contextualised evidence. This formative research study design will provide a contextual evidence-base including patient perspectives. This can be used to underpin the collaborative co-design of a multidisciplinary, blended model of care leveraging efficient and cost-effective care services suited to patient preferences.

## Introduction

Cardiovascular diseases are responsible for a third of all deaths globally. In developed countries such as the USA and Australia, 5–6% of the adult population report a heart disease diagnosis, most commonly coronary artery and valvular disease, with the prevalence increasing with age [1,2]. The association of psychosocial conditions with cardiac disease development, adverse medical and mental health outcomes and increased healthcare costs is well established [3,4]. In hospitalised patients awaiting cardiac surgery, the incidence of patients meeting criteria for major depressive disorder is reported to be 20–40%, with preoperative anxiety at over 30%, increasing up to 45% following cardiac surgery [5]. Cardiac surgery is experienced as a significant life event [6] with mortality dependent on variables such as procedure, patient co-morbidities, and unit surgical volume, reported to range from 0.4% (e.g., low-risk Swedish coronary artery bypass graft cohort [7]), to over 21% in aortic dissection surgery (US cohort, [8]). Risk models including up to 40 physiological factors predictive of mortality in cardiac surgery, have been in use since the 1980s [5] with the significant contribution of emotional, behavioral, social, and functional factors to outcomes increasingly recognised [5].

### Psychosocial measures as risk factors in cardiac surgery

Meta-analyses show depression independently predicts higher operative early and late mortality after cardiac surgery (both Relative Risk Estimates, RRE 1.44), while perioperative anxiety has been found to predict increased late mortality (RRE 1.81) [9] and combined postoperative mortality and major morbidity (Odds Ratio, OR 5.1, confidence interval (CI) 1.3–20.2 [10]). This is similar in magnitude to that of physiological risk predictors for postoperative mortality such as chronic pulmonary dysfunction (OR 1.9, CI: 0.9–1.5), myocardial infarct (OR 1.2, CI: 0.9–1.5), or diabetes (OR 1.4, CI: 1.1–1.9) [11].

Increased post-operative morbidity and health service costs are also associated with perioperative psychological factors [5]. More specifically, Oxlad et al. [12] estimated 24.5% of variance in morbidity outcomes is attributable to medical parameters, with psychological factors contributing an additional 4.4%. AbuRuz et al. [13] found a 1-unit increase (Hospital Anxiety Depression Scale) in preoperative anxiety increased length of stay (LOS) by 0.38 days on average. Related findings include an increased risk of major adverse cardiac events and health service utilisation, poorer Quality of Life (QoL) [14–16], and a 2–3-fold increase in 6-month readmission risk [17]. Conversely, positive emotions may reduce readmission and mortality [18]. Potential explanatory psychobiological mechanisms mediating the effects of psychological state on operative outcomes include both behavioral pathways (e.g., medication adherence, lifestyle choices [18,19], and biological mechanisms (e.g., inflammatory processes, dysregulation of the hypothalamic–pituitary–adrenal axis, reduced heart-rate variability), [9,19] although no specific laboratory tests provide a direct and reliable means of assessing such psychological status [20], particularly in the perioperative context.

### Limitations in measurement of psychosocial risk

Detection and management of psychosocial risk factors offer a potentially valuable opportunity to improve health outcomes for cardiac surgery patients. However, methodological limitations in many studies to date, such as inadequate methods used to detect risk factors and adjust for potential confounders, undermine their ability to inform effective clinical management strategies [5,21]. The variability of reported prevalence and impact of psychosocial health exposures in coronary artery disease (CAD) and surgery, compels a careful psychological profiling of target patient populations and exploration of the relationship with outcomes relevant to local system settings. This pertinent knowledge can then inform tailored options for future screening and management implementations and enable nuanced evaluation of their effectiveness [22,23].

Kohlmann [22] found patient variables and system level factors are likely to have influenced detection of psychosocial risk in previous studies and need careful evaluation in the guidance of implementation strategies. For example, the size of the reported association of depression with post-operative mortality is inconsistent with contrasting findings between various large retrospective population-based linkage studies, prospective nested cohort studies and meta-analyses [24]; adjustment for confounders, such as physiological risk scores and relevant social variables, amongst these studies is often incomplete and case identification methods vary. For example, Stenman et al. [25] used retrospectively ICD-coded diagnoses to identify depression while others used prospective self-reported instruments such as the Hospital Anxiety and Depression Scale [24], with differing covariates used to account for confounding and various surgical procedures across cohorts. Furthermore, moderators may impact the relationship with outcomes, potentially accounting for some of the observed variability in effect measures in the literature. Influential moderating or confounding covariates may include sex, personality traits, stress, anger, hostility, social isolation, and occupational stress which have been shown to feature consistently as influential in myocardial disease progression [26,27], while perceived control may moderate the effect of anxiety with post-operative LOS [13].

### Considerations in management of psychosocial risk

Despite an imperfect understanding of preoperative psychosocial predictors on cardiac surgery outcomes, psychological interventions following a diagnosis of CAD, myocardial infarction or revascularization have demonstrated effectiveness in improving psychological symptoms and reducing cardiac mortality [28]. Nevertheless, uncertainty remains regarding the effectiveness of specific techniques most likely to benefit patients under differing contexts [28,29] and the associations between patient preferences in peri-operative management and key surgical outcomes [30]. Furthermore, the acceptability of depression screening and patient preferences for mental health care [18] still need clarification, especially around barriers such as doubts about screening validity and stigma [31].

Reuter [32] finds patients and healthcare professionals to be the two key stakeholder targets for improving uptake of screening and referral guideline implementation, concluding a multi-level (system and patient) theory-informed approach is needed. However, peri-operative care guidelines development has generally failed to incorporate patients’ unique insights such as about therapeutic burdens, unmet needs, balance of benefits and risk, which could aid in the success of system change processes [30]. Ultimately behavior change in stakeholders is required for a screening program to be effective, contingent on recognizing and addressing patient needs and barriers, and tailoring the program to patient and societal preferences and concomitant health system issues [22,33].

Screening alone is insufficient to ensure adequate psychosocial risk management but is widely recommended as a critical step in improving cardiac patient outcomes [22,26,34]. Yet screening and management strategies targeting depression have been poorly implemented and the burden of depression in patients with cardiovascular disease remains under-recognized [35]. Prospective psychosocial risk management and psychobehavioural optimization feature in surgical prehabilitation programs [29] and align with ERAS (Early Recovery After Surgery) protocols, a multidisciplinary approach aiming to prospectively reduce physiological and psychological stress in surgical patients to improve recovery [36]. Although prehabilitation trials began over 2 decades ago, recent review confirms an inconsistent approach to the psychobehavioural component and a stronger focus on the physical and nutritional aspects in cardiac surgery [29]. The implementation of ERAS protocols in cardiac surgery is still an emerging field with relatively low-quality evidence available on recommended interventions [37]: notably, systematic psychotherapeutic care structures in cardiac surgery are currently lacking [6]. Moreover, the efficacy of proposed psychosocial interventions following screening is subject to a variety of barriers and contextual factors such as intervention type, target population, and study characteristics [38], complicating intervention selection and implementation.

Cost and resource implications of integrating psychological treatment must be considered alongside any screening program [39]. Promising innovative interventions such as blended care utilizing eMental Health (eMH) online platforms and a tailored approach may offer cost-effective management strategies [40], noted as effective for patient engagement in cardiac surgery ERAS studies [36]. However, further research using theory-led approaches is recommended to understand facilitators and barriers for the implementation of such innovative services into health care [41].

### Implementation science in management of psychosocial risk

Recently implementation science theories have been employed to investigate psychosocial screening uptake in CAD [23]. Barriers are context-relevant determinants impeding implementation, while facilitators promote the implementation, each operating on various levels, such as external, organizational, professional, and interventional [23]. Relevant service level barriers include time constraints, disruption of primary activities, insufficient integration of mental health resources, and limited knowledge on psychosocial risk and screening [23,39]. Patient barriers were commonly related to accessibility and patient characteristics such as stigma and acceptability of mental health treatment, although patients were generally more positive to screening than were health professionals [22,23]. Before designing and implementing an intervention, formative research can be used to explore stakeholder behaviours, perceptions, and factors that may influence program success or failure, with the aim of reducing potential barriers [42].

Using a theory informed Knowledge to Action framework [43], the EMBRACE study (Exploring the relationship between EMotional well-Being with health outcomes and patient pReferences for resources and support in cArdiaC surgEry) will undertake formative research aiming to develop the knowledge to underpin collaborative co-design of an evidence-based psychosocial screening program in cardiac surgery.This formative research employs a pragmatic research and informatics approach [44,45] to synthesize and tailor knowledge to the real-world setting, developing contextual evidence and knowledge products, and considering patient and health service priorities. The EMBRACE study focuses on defining the local psychosocial contribution to patient outcomes and patient barriers but is the first part of a research program that also plans to examine concomitant implementation issues including health professionals’ perceptions, needs and preferences regarding psychosocial screening and management. Such development of contextual evidence can bridge the gap, creating awareness across health-care professionals’ and patient stakeholders’ preferences to better guide future collaborative co-design of potential solutions to improve outcomes through the peri-operative care episode [30]. Also, given many post-surgical patients have increased psychological comorbidity for at least 6 months, and that neglecting these needs has significant implications for health service costs [17], assessment of health outcomes beyond discharge is critical to informing the effectiveness and sustainability of innovative interventions such as blended care, from the health system perspective. This study is expected to provide additional knowledge in how to guide development and implementation of psychosocial screening and interventions by answering the research question: What is the prevalence and impact of psychosocial comorbidity across the spectrum of cardiac surgical patients, and how do patient preferences inform the design of a practical psychosocial screening and support program in a public hospital setting?

### Study aims and objectives

The EMBRACE study aims to: 1. characterise the baseline prevalence of self-reported psychological comorbidity within an inclusive Australian sample of public patients receiving a broad spectrum of cardiovascular surgery, 2. define their relationship with post-operative health outcomes to 30 days and 12 months, accounting for potential moderators and confounders, and 3. explore patient preferences for their emotional and mental healthcare screening and support, including eMH resources, and relationship to their clinical and psychological profile.

The specific objectives of EMBRACE study are to:

1. Estimate the baseline prevalence of key psychological comorbidity in relation to:
  i. Depression measured by the Patient Health Questionnaire – Depression (PHQ-9)
  ii. Anxiety measured by the Generalized Anxiety Disorder 7 scale (GAD-7)
  iii. PTSD measured by The Primary Care PTSD Screen for DSM-5 (PC-PTSD-5).
2. Assess the association between psychological co-morbidity risk factors and post-operative health outcomes adjusting comprehensively for physiological risk, demographic, social, and psychological functioning covariates:
  i. Days alive and out of hospital at 30 days (DAOH; primary outcome)
  ii. Composite major adverse events at 30 days (MAE), all-cause mortality and readmission at 12 months, health-related quality of life measured by EQ-5D psychological co-morbidity, and total costs for admission and readmissions to 12 months (secondary outcomes).
3. Investigate and explore patient preferences regarding psychosocial screening and support by:
  i. Ascertaining patient interest and preferences for feasible psychosocial supports as measured by a novel patient preferences survey
  ii. Exploring patient characteristics and psychological risk factors associated with these preferences
  iii. Ascertaining the comprehensiveness of support options offered by the novel patient preferences survey and any further support suggestions by sub-group participant semi-structured interview.

## Methods

This research protocol paper conforms to reporting requirements according to GUIDED – a guideline for reporting for intervention development studies checklist [46] (see S1 Checklist).

### Study design

This study will investigate the research topic through contextual enquiry, exploring the potential contribution of psychosocial variables to cardiac surgery patient health outcomes and patient preferences for their emotional and mental health aspect of care in a single large public hospital setting. It will use a concurrent mixed methods approach to assess a potential gap in clinical care processes. This firstly will incorporate a quantitative prospective observational cohort study utilising patient screening instruments for psychological and QoL measures, linked with electronic record, clinical information systems and medical chart review (where necessary) for physiological risk and surgical and health service outcomes.

Secondly, a brief novel survey will be used concurrently to elicit patient preferences for mental health care according to their individual psychosocial and clinical profile, together with limited semi-structured interviews of a purposively selected patient subset. This will be an explanatory-confirmatory concurrent design where the supplementary interview qualitative data will be used to explain and validate the core quantitative preferences survey results and identify potential omission of support options offered in the survey, as well as elicit any additional thoughts participants may wish to offer to provide a succinct but thorough understanding of patient perceptions on feasible support options.

### Study setting

The research will take place within the cardiac surgical program at The Prince Charles Hospital (TPCH), a large public quaternary level cardiac centre in Brisbane, with follow-up occurring via electronic survey, phone call, postal survey or the Outpatients clinic. The Prince Charles Hospital performs one third of total Queensland cases and close to half of public cases [47] with 985 procedures performed in 2021, a slightly reduced volume due to COVID-19. Before the pandemic, elective cases and urgent cases comprised 33% and 58% of the cohort respectively, with less than 1% under 18 years of age. The program patient case-mix includes complex acquired and adult congenital heart surgery cases. All cardiac surgical patients booked for surgery (i.e., elective or urgent) will be considered for eligibility in the study. Therefore, we expect over 900 cases annually providing an adequate sampling population for recruitment of 260 cases in 12 months.

### Participants

#### Inclusion criteria.

All adult cardiac surgery patients will be eligible for participation if meeting the following inclusion criteria:

- of adult age (>= 18 years);
- booked for elective or urgent surgery and planned to progress to cardiac surgery within 1 week of recruitment and within the study timeframe;
- able to be recruited and assessed no less than one day prior to surgery;
- available in a routinely accessible ward by an available investigator;
- capable of understanding the Participant Information and Consent Form (PICF) and provide signed consent;
- able to provide responses to the psychosocial screening tools and the participant preferences survey, with researcher support if required.

#### Exclusion criteria.

Exclusion criteria will consist of:

- contraindicating advice of the clinical care team prior to approaching the patient. This includes: (a) prior indication that the patient is not interested in participating in research, (b) individual surgeon’s preference that their whole patient cohort is not included or (c) clinical concern that a patient may be experiencing significant behavioural or mood disturbances that could be exacerbated by psychosocial assessment.

Patients may be excluded at the discretion of senior ward nursing staff if they are deemed emotionally labile or at risk of undue distress from participation and being unlikely to derive any benefit from participation. This exclusion is ethically justified under the NHMRC National Statement on Ethical Conduct in Human Research, 2023 [48], which requires researchers to minimize harm and justify exclusions of at-risk individuals. From previous experience we expect fewer than 1% of patients are likely to be excluded under this criterion and any associated potential bias to be minimal. Any potential bias will be assessed by comparison of baseline characteristics.

### Measures and instruments

Table 1 summarises measures, data sources and timeframes for data collection. A panel of psychometric instruments were chosen for predictor variables and potential important covariables and moderators, based on evidence of contribution to cardiac surgery outcomes and/or cardiac disease progression, similar to the core questions for psychosocial risk assessment recommended by Germano et al. [49]. From a pragmatic perspective, measures were selected to be feasible and actionable [45]; instruments were selected for brevity, ease of use, and to minimize burdens to patients and staff during the busy perioperative period [5], being intended as a screening rather than diagnostic tool. The main case identification instruments (anxiety, depression, PTSD) were chosen for strong psychometric properties and proven validity in cardiac populations. Alternatives such as diagnostic interviews or panels of physiological markers are not feasible for scalable screening programs.

**Table 1:** Variables, data sources and timeframes for data collection. Definitions may be found in S1 Variables definitions.

Variable group and collection timepoints	Data Source and variables
Psychometric variables and additional socio-economic variables at baseline	Demographic covariables • Income level • Education level
	Social and relationship covariables • Living alone (number of cohabitants) • Attachment Style-Relationships as Secondary (ASQ-SF 4-item sub-scale) [50]
	Pre-existing mental health conditions • Previous use of psychological support services • Previous mental health diagnosis – If yes: • Current or recent medication for mental health condition • Cognitive function –Clock Drawing Test and 3-word verbal recall [51]
	Psychological coping covariables • 4-item Brief Resilience Coping Scale (BRCS) [52] • 6-item Life Orientation Test Revised (LOT-R) [53] • Perceived Stress Score (PSS-4) [54]
Psychological co-morbidity variables • baseline • 1 week/discharge • 6–12 weeks postop • 1 year postop.	Psychometric exposure measures and psychological functioning • Patient Health Questionnaire – Depression (PHQ-9) [55] • Generalized Anxiety Disorder 7 scale (GAD-7) [56] • Primary Care PTSD Screen for DSM-5 (PC-PTSD-5) [57] • New psychological support services • New mental health diagnosis – If yes: • New medication for mental health condition
Quality of Life scale at • baseline • 6–12 weeks postop • 1 year postop.	• EuroQual measure of health-related quality of life (EQ-5D-5L) used in a wide range of health conditions and treatments [58]
Patient Preferences for emotional and psychological care management at • 1 week/discharge	Survey of patient preferences and interest in emotional well-being or mental health support including: • Interest in mental health/ well-being support during cardiac surgery episode • Additional patient education videos and reading material • On-line relaxation supports and resources • On-line coping and psychological supports and resources • Peer patient volunteer contact and support • Health professional consultation
	Semi-structured interview of preferences for mental health care (nested sub-group)
Cardiac Surgical outcome related covariates routinely collected in electronic data systems	QCOR, ICU, QHAPDC, QNAPDC data extraction including: • **Demographics:** • Age, sex, Indigenous status • **Surgical risk and perioperative covariates:** • urgency status • surgical risk score (EuroSCORE II) • surgical procedure (CABG, valve, other) • surgical procedure complexity (isolated, multiple or complex) • Perioperative covariates (bypass time, blood product use, mechanical support requirement, ICU Delirium Score) • **Primary Outcome:** • DAOH -Days Alive and Out of Hospital at 30 days (incorporates LOS and readmitted days subtracted from 30 days, in-hospital death = 0) • **Secondary Outcomes:** • In-hospital Major Adverse Event (MAE) composite of the following: CVA, Renal Failure, Ventilation >24 hours, Reoperation, Deep Sternal Wound Infection • Length of Intensive Care Unit stay (ICULOS) • Length of ventilation • New atrial arrhythmia requiring treatment • Delirium • Re-admission within 30 days and reason • Attendance at cardiac rehabilitation services • readmission within 12 months • Mortality (all cause) within 12 months • Total costs for admission and readmissions to 12 months.

Legend: QCOR: Queensland Cardiac Outcomes Register, ICU: Intensive Care Unit, QHAPDC: Queensland Hospital Admitted Patient Data Collection, QNAPDC: Queensland Non-Admitted Patient Data Collection, LOS: length of stay, CVA: cerebrovascular accident.

The surveys comprise self-complete instruments for depression, anxiety, PTSD, coping, life orientation, attachment style, perceived stress, and health related quality of life. Socio-economic variables include income level; education level, living alone, and a brief cognitive function assessment will also be included.

Physiological covariates and outcome measures routinely collected and used in cardiac surgical quality monitoring and reporting are defined according to the Queensland Cardiac Outcomes Registry [47]. Cause of death data will be obtained by linkage from the Queensland Births, Deaths and Marriages Registry after 1 year. Costs and health service utilisation associated with the case during admission and up to one year after surgery will be extracted from Queensland Health clinical and administrative systems via data linkage (Queensland Hospital Admitted Patient Data Collection, QHAPDC, Queensland Hospital Non-Admitted Patient Data Collection, QNAPDC).

The primary outcome measure for assessment of the effect of patient baseline psychometric status detectable by the screening instruments is Days Alive and Out of Hospital in 30 days (DAOH). This incorporates post-operative LOS and readmitted days subtracted from 30 days, with hospital death = 0 and has been recommended as a sensitive easily quantifiable patient-centred quality outcome measure in cardiac surgery [59,60].

Secondary outcomes of interest include in-hospital MAE composite (CVA, Renal Failure, Ventilation >24 hours, Reoperation, Deep Sternal Wound Infection) as defined by QCOR [47], postoperative LOS, length of Intensive Care Unit stay (ICULOS), delirium, re-admission within 30 days and reason, attendance at cardiac rehabilitation services, readmission within 12 months, death (all cause/cardiac) within 12 months, total costs for admission and readmissions to 12 months (definition details provided in S1 Variables).

Assessment of patient preferences and associations with psychosocial exposures of interest provides an opportunity to tailor a screening and intervention program to individual needs. An understanding of attitudes and acceptability for psychosocial supports will be drawn from a questionnaire designed to present potential feasible options available in the context of an Australian public health service including online eMH supports while a confirmatory brief interview component adds a level of assurance to the comprehensiveness of the positivist survey component [61]. Being primarily exploratory, the survey was designed with a parsimonious validation process, i.e.,

- Expert and stakeholder question design: Via expert psychologist (academic and service director) contribution and consultation with service staff stakeholders (nursing, medical, allied health)
- Consumer Liaison review: To assess clarity, relevance, and usability.
- Face validity and content validity assessment: semi-structured interviews with a patient subgroup to ensure all relevant support types are included and none are misleading or ambiguous.

While the survey was not intended to measure a latent construct and therefore not subject to full psychometric validation, reliability statistics and internal consistency with Cronbach’s alpha will be reported in a future results paper. Further reproducibility testing may be considered in future implementation phases if required for different contexts.

**Fig 1:**
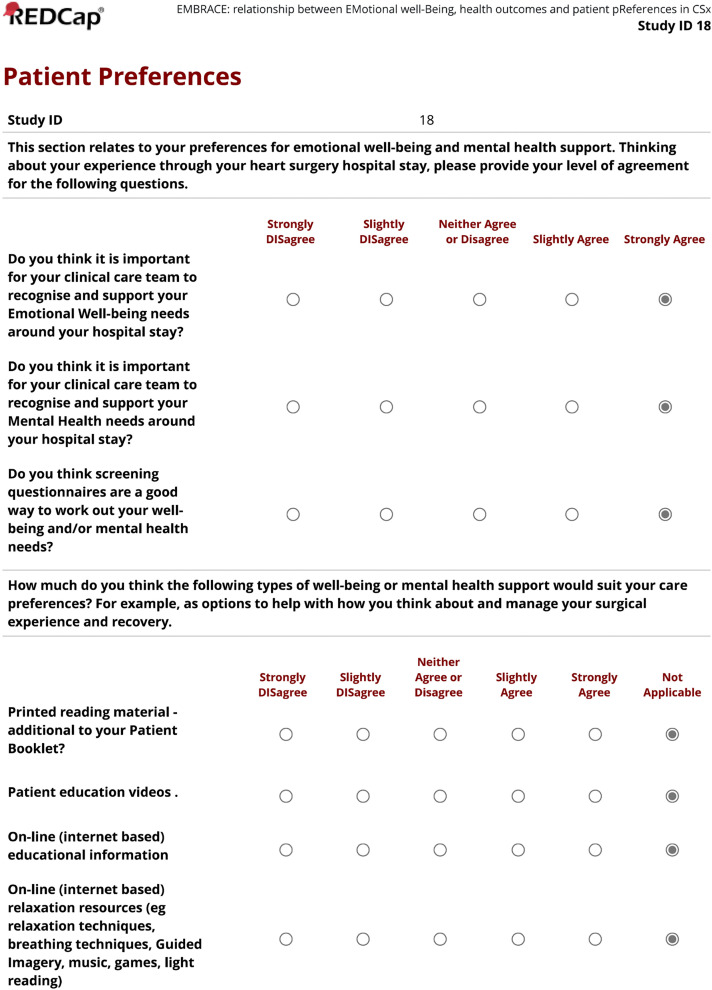
Patient preferences for psychosocial supports survey.

The options presented for participant evaluation in the survey reflect an escalating stepped care response – and increasingly resource intensive – options for psychosocial support needs ranging from additional reading in existing patient education material, to health professional organised individual psychosocial supports plans following discharge (Fig 1). The patient preferences survey will be aggregated and analysed for a) participants’ perception of the importance of emotional well-being in the cardiac surgery episode of care (first 3 questions) and b) their interest in use of the suggested psychosocial support options (last 8 questions), using a 5-point Likert scale (scoring 1–5). For those participants who do not have access to or capacity to use technology supportive of eMH options ‘Not Applicable’ is provided as a response.

### Sample size estimation

#### Psychosocial exposure variable prevalence.

For the baseline prevalence estimates for depression and anxiety, a sample size of 260 produces a two-sided 95% CI with a width equal to 0.10 when the sample proportion is 0.20, which is in the order of proportions reported elsewhere and would be sufficient to confirm presence of raised mental health conditions in the cohort compared with reported normal population rates of around 5% each (PTSD approximately 1%) [62].

#### Primary outcome.

Days alive and out of hospital to 30 days is routinely available for all patients. In a recent study of 304 patients at TPCH, 18 (6%) had scores of zero and 77 (25%) spent longer than 10 days in hospital. A proposed sample size of 260 achieves 80% power to detect an effect size (f²) of 0.048 attributable to 4 independent variables using an F-Test with a significance level (alpha) of 0.05. The variables tested will be adjusted for an additional 5 independent variables. The calculations assume an unconditional model (PASS [63]).

#### Secondary outcomes.

Post-operative MAE composite contributes to poorer long-term outcomes after cardiac surgery. Approximately 15% of Queensland public cardiac surgery patients experience any MAE [47]. For 260 cases a rule of thumb formula of n = 100 + 50i (where “i” refers to the number of independent variables), suggests 3–4 independent variables may be included reliably in a final multivariable logistic regression model [64] allowing determination of important predictors of outcome through successively testing a number of models based on theory and findings from the linear regression analysis of DAOH.

The main long-term outcomes will be all-cause mortality and resource utilisation at 1 year according to exposures of interest. For an anticipated loss-to follow-up of 20%, 208 of the proposed 260 patients would remain with sufficient data for inclusion for follow-up analysis. Given the small sample, analysis of longitudinal health outcomes to identify potentially distinct trajectory sub-groups will be exploratory.

The above considerations suggest a patient cohort recruitment target of 260 would provide an acceptable level of power for estimation of prevalence of mental health exposures of interest and prediction of DAOH and MAE outcomes and health service resource utilization to 1 year.

#### Patient preferences.

The quantitative patient preferences survey will be given at discharge to all participants and the estimated loss to follow-up of 20% provides 208 results for a descriptive and exploratory analysis of participant interests for support options offered.

The qualitative semi-structured interview sub-sample is a convenience purposive sample, aimed at providing a cross-section of participants for sex, age, ethnicity, procedure complexity, preoperative psychological status and if participants indicate an interest in additional study contribution. It is estimated to require a small number of approximately 10 as guided by the Information Power criteria [65] i.e., a narrow aim to explain and confirm the referent preferences survey and identify potential gaps in psychosocial support options; limited participant specificity; use of an established stepped care framework underpinning the referent preferences survey; expected good quality dialogue based on investigator experience and knowledge and an intention for a limited pragmatic deductive thematic rather than exhaustive exploratory analysis.

### Procedure

**Fig 2:**
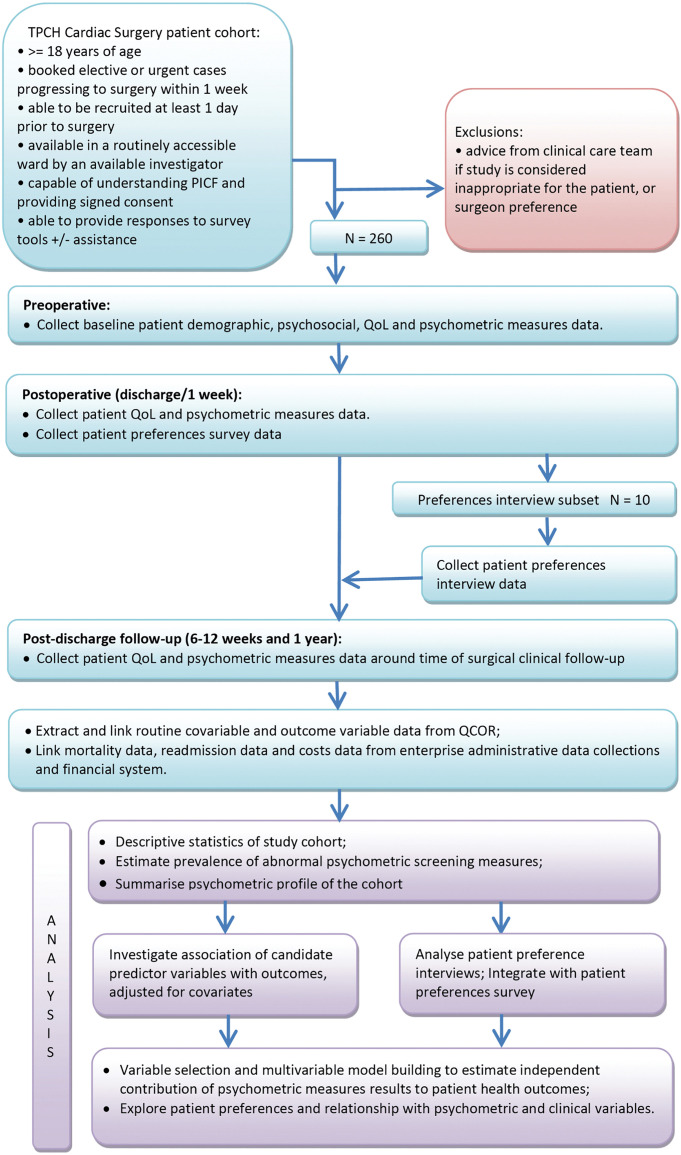
EMBRACE study design and data collection flow diagram.

The study has already gained support and engagement from relevant clinical stakeholders including: cardiothoracic surgeons and anaesthetists via departmental meetings; clinics and wards, including the coronary care unit, cardiothoracic surgical ward, outpatient clinic and surgical bookings office by consultation with the Medical Directors and Nurse Unit Managers; hospital social work and psychology services by consultation with the service directors and engaged staff; and a hospital patient consumer representative. The study design and data collection flow diagram are shown in Fig 2.

#### Participant recruitment.

All elective adult cardiac surgery patients will be sent an invitation letter (S1 Letter) with a brief description of the study together with the routine documentation sent by the Elective Surgery Coordinator on booking for surgery. This allows patients to be made aware of the study through their care team before admission. On admission and booking in the theatre system, potential participants will be pre-screened for inclusion criteria via the clinical information system and admitting staff (age, known communication or comprehension difficulties, < 1 day to surgery, ward clinician consultation). Suitable patients will then be approached, invited to participate, informed of the EMBRACE study details and provided with the PICF (S1 PICF) by an appropriately trained and delegated member of the research team, informed they may withdraw at any time and may have any study information about them destroyed if wished with no consequences to normal care. Following explanation of the study, written consent will be sought from the patient and if consented, participant inclusion and exclusion criteria reviewed.

Urgent adult cases booked in the theatre system potentially suitable for recruitment with pre-surgical hospital stay greater than 1 day are provided by the theatre bookings office and will be pre-screened as for elective cases. The accepting ward staff will then introduce the researcher prior to providing the patient with the study information, PICF and being consented if agreeable, as for elective patients.

#### Data collection.

For enrolled participants, an appropriately trained and delegated member of the care or research team will facilitate collection of the self-reported screening data using the secure web-enabled REDCap platform [66] on a mobile tablet, on admission or while awaiting surgery in the ward. Participants will be provided with assistance in answering the survey instruments if required, including reading the questions and entering answers on the device if necessary.

At discharge, the postoperative psychometric measures will be collected and participants will also be provided with the patient preferences survey and a convenience sample subset will be invited to participate in a short semi-structured interview guided by a script (S1 Script) to ensure the patient preferences survey is adequate and to elicit any further participant preferences which may have been otherwise missed. This interview will be conducted by telephone in the early post-operative period. Consent for interviews is included as part of the study consent at recruitment, and at interview participants’ agreement to the additional interview will be confirmed and their consent recorded in the audio file. At the end of the interview, participants will be asked to reflect on their input and if they are satisfied with their responses.

Participants will be again provided with the screening instruments at 6–12 weeks timed around the first post-operative clinical review, and at the one-year review, to approximately coincide with clinic visits, avoiding burden on the participant to return at extra times and aligning with recovery phases. During recruitment, the participant will be firstly offered email contact with a secure web link to report their post-operative status directly to the study database or alternatively offered a phone call or a postage paid return addressed envelope to complete their follow-up information or met at Outpatient clinic follow-up. If a participant is discharged before the second survey can be completed face to face, the participant can be contacted by phone and invited to complete the surveys online via a secure link or verbally by phone.

#### End of study.

The last survey at one year includes a message thanking participants and providing them with an option to indicate if they would like to see the results of the study and the participants are provided with opportunity to provide feedback on the study throughout the survey collection points.

### Data and analysis

#### Data management plan.

Psychometric factors are measured with self-reported questionnaires, provided in the ward via mobile tablet technology at baseline and discharge and via secure online link or phone call/letter/OPD clinic at follow-up. Psychometric screening data is directly captured into the research database using REDCap, a mature, secure web application for building and managing online surveys and research databases [66], provided within the Queensland Health secure digital environment. Preoperative medical and physiological risk, procedure, outcomes, and administrative data will be extracted from relevant sources as described and linked within the Queensland Health enterprise services, according to Queensland Health policies regulating the secondary use of confidential information.

Participant interview data will be collected via telephone and utilise Microsoft Teams recording and automated transcription and be stored in a secure Queensland Health approved file location. Automated transcription will be reviewed against the recordings and corrections made by the interviewer.

Access to identifiable data will be limited to investigators and only as necessary and will be de-identified prior to analysis after linkage, retaining a linkage key for potential data quality resolution.

#### Analytical plan.

Continuous data will be presented as mean (+/-SD) or median (IQR) as appropriate; categorical variables will be described as number and percentage. The distributions of data will be evaluated and assumptions for statistical analyses assessed, and transformations will be applied if required. Psychometric screening instrument scores will be calculated per case and summarised as sample means with 95% CI. Scores indicating elevated risk of moderate depression, anxiety or PTSD will be ascertained according to current recommendations (Table 2) [56,57,67]. The prevalence of abnormal scores for the mental health exposures of interest (Depression, Anxiety, PTSD) will be estimated as proportions with 95% CI. Continuous measures between groups will be compared using Student’s t test, ANOVA, or non-parametric tests as appropriate; categorical group differences will be tested with Chi-Squared tests. Regression models will report regression coefficients, Relative Risk or Odds Ratio with 95% CI, p-values and F-statistic with incremental R^2^ for psychosocial exposures of interest. Goodness-of-fit tests and model-fitting diagnostics will be performed for proposed analyses assessing for influential points, outliers, and to evaluate alternative model specifications. All hypothesis tests will be considered with significance level alpha = 0.05. Statistical analyses will be conducted using SPSS Statistics (IBM 2023) and Stata, (StataCorp, 2023).

**Table 2:** Psychometric instrument scores used for case identification.

PHQ9 Score	Depression severity score inclusion
10-14	Moderate depression
15-19	Moderately severe depression
20-27	Severe depression
**GAD7 Score**	**Generalised anxiety severity score inclusion**
10-14	Moderate Anxiety
15-21	Severe Anxiety
**PC-PTSD-5 Score**	**PTSD severity score inclusion**
4-5 (males)	Probable PTSD
3-5 (females)	Probable PTSD

Legend: PHQ9: Patient Health Questionnaire – Depression scale; GAD7: Generalized Anxiety Disorder 7 scale; PC-PTSD-5: Primary Care PTSD Screen for DSM-5.

**For Objective 1:** Participant characteristics will be described and compared against a) the entire cardiac surgical population, b) clinician advised exclusions and patients declining to participate for reasons potentially related to emotional concerns (as recorded on a Participant Tracking leger) as a potential indicator of sampling bias. Estimation of depression, anxiety and PTSD will be made as point prevalence percentage rates of moderate-severe depression, moderate-severe anxiety, or probable PTSD (Table 2) in the study cohort at the indicated time points.

**For Objective 2:** Associations between mental health exposures with DAOH and other continuous secondary outcomes, will be analysed using univariable and multivariable linear regression analysis. Models to estimate the independent contribution of the candidate predictor for outcomes will be adjusted for potential confounders including age, sex, EuroSCORE II, procedure, surgical complexity and urgency status. Heterogeneity will be explored by testing interaction terms during model building of potential moderators including status, procedure, complexity, Coping style, Life Orientation, Perceived Stress, Attachment Style, mental health diagnosis, cognitive function. If there is evidence of heterogeneity with sufficient numbers per subgroup, subset analyses may be conducted. Exposure variables may be categorised using clinically meaningful cut-points.

Binary secondary outcomes of interest (MAE) will be analysed using logistic regression analysis. The contribution of components in composite outcomes such as specific morbidities, LOS or readmission, may be considered in the model-building if exploratory analysis indicates potential utility. Potential confounders and moderators will be tested in models.

Changes in psychometric measures over time will be explored using mixed effects linear regression modelling. This method uses all available data and allows inclusion of all patients, even if follow-up is incomplete, provided they have data for at least one time-point. The effects of baseline patient characteristics (e.g., age, sex, status, EuroSCORE) will be explored and adjusted predictions at each time-point will be used to determine the predicted prevalence of each condition (anxiety, depression, PTSD) at time-points of interest. The effects of incomplete follow-up will be assessed by comparing these predictions to the observed proportions amongst those with follow-up data. One year survival will be described using Kaplan-Meier methods and assessed using Cox Regression survival analysis if sufficient events occur in the cohort to warrant analysis.

Associations between mental health exposures (Depression, Anxiety, PTSD) and total health economic costs to 1 year will be undertaken from the perspective of the public healthcare system to estimate the potential for cost savings from an effective screening and intervention program. Direct health care costs in terms of utilization of health care resources (LOS, readmission, re-presentation to Emergency Department) and associated monetary costs will be deduced for participants in relation to exposures of interest.

**For Objective 3:** Survey responses will be summarised and associations between survey responses and psychological measures will be explored using regression analyses as appropriate. Interview results will be analysed using a deductive thematic analysis approach [68] in relation to the quantitative patient preferences survey, according to a) perceptions of emotional and mental health needs in cardiac surgery, b) preferences for support, including eMH options, c) comprehensiveness of the patient preferences options for support as provided on the preferences survey. The qualitative results will be used to complement the quantitative results to identify any omissions in the survey for support preference options [69] and test the underlying assumptions about provision of psychosocial screening for cardiac surgical patients. The results of the quantitative and qualitative analyses will be synthesized to contextualize patient preferences in the Australian public health service.

### Ethics, governance, and safety

The Metro North Human Research Ethics Committee B approved the study as Low Risk Research, HREC/2022/QPCH/80524 and all participants will provide informed signed consent prior to data collection. A waiver of consent was also approved for extraction of de-identified data from non-participants for comparison. The study will comply with the approved protocol, ethics approval conditions, the NHMRC National Statement on Ethical Conduct in Human Research, 2023 [48] and the Australian Code for the Responsible Conduct of Research, 2018 [70] and all relevant national and state legislation and Queensland Health privacy and confidentiality standards.

As an observational study with no embedded referral process, participants are informed during recruitment that the study offers no direct personal benefit, aside from encouraging reflection on emotional well-being, while the potential of emotional triggering from anxiety and depression screening is described as a low but plausible risk for some patients. Participants will self-report psychometric measures directly into a research database without prospective clinical review of responses. However, if the participant discloses information to the researcher that may impact their care or requests to speak with a care team member about concerns identified during the study, professional duty of care requires referral to the routine care team. Any new patient risks identified during the research process will be communicated to the care team via standard clinical procedures. This includes consultation with the ward nursing staff first, communicating with the consultant surgeon if necessary for referral, followed by escalation to the cardiothoracic service medical director and submission to the Queensland Health Riskman system used to log clinical and non-clinical incidents for formal Quality and Safety review if required. Any adverse events arising from the research study itself will be reported to the hospital Research Office according to State and National guidelines.

Metro North Health Service is the study sponsor and governance procedures dictated by the Metro North Research Office will be followed. Study site files will retain a record of screened and invited patients, responses, signed PICFs, inclusion/exclusion criteria checklist and record any unexpected or adverse participant reactions related to the conduct of the study, which will be reported to the clinical care team and the Metro North Research Office as relevant.

### Study status

At the time of writing 219 participants have been recruited and follow-up surveys are on-going with the last follow-up to occur 1 year after the date of the last participant’s surgery.

## Discussion

This formative research is intended to provide a contextual evidence base for the subsequent co-design of a collaborative, blended model of care leveraging cost-effective care services suited to individual patient needs. As published evidence on successful screening program implementation remains inconsistent, a re-evaluation of the evidence within the local context is necessary and this research addresses key target actors including patients and the health service perspective to contribute to the evidence base. Quantifying the prevalence and impact of independent psychosocial risk factors is a necessary knowledge product for driving development of an appropriately calibrated screening and intervention program.

Following model building, significant psychosocial measures will be considered to identify a practical set of instruments for a screening panel targeting modifiable psychosocial risk factors linked to key surgical outcomes. The intention is to efficiently identify at-risk patients who may benefit from additional psychosocial support. Understanding patient needs and preferences for screening and support is essential for designing a successful tailored care approach and will contribute to the limited existing evidence, including current acceptance of screening and use of eMH resources.

One-year outcomes will provide information relevant to patients’ longer term quality of life and health service utilisation as a baseline target for improving health and resource utilisation outcomes. As this protocol is the first part of a larger translational research program, healthcare professionals’ perspectives and potential barriers will be further elucidated in the next phase of research co-designing and trialling a new model of care based on the findings of the EMBRACE study.

### Strengths and limitations

Strengths of this research include a pragmatic data-driven informatics approach which leverages existing information resources such as real-world clinical information systems and spurs adoption of mobile technology as an operational tool. The study design prioritises minimising burden on both patients and health services, while addressing gaps or inconsistencies in the extant evidence, such as prevalence of psychosocial risk factors amongst the wider surgical cohort and their independent effect size on outcomes. It will also extend what is known about confounders and potential effect moderators. Patient selection will be inclusive to ensure implementations are relevant to the full spectrum of patients and surgical procedures experienced in a large public hospital, whereas many other studies are limited to subgroups such as coronary artery bypass patients.

Patient preferences will be elucidated as an integral part of the research study, enabling exploration of preferences according to psychosocial exposures of interest as well as relevant medical and demographic variables. A novel patient preferences survey was developed including support options gathered from healthcare professional’s suggestions and patient advocate input. While this is not a validated instrument, it offers available options according to the stepped care model of psychosocial support [71,72], including interest in eMH supports and results will be verified against a small subset of participant interviews. This will be an exploratory investigation to gain an initial understanding of patient interests and perspectives and to formulate a more thorough investigation as part of future service co-design if indicated.

The EMBRACE protocol addresses pre-operative psychosocial screening in cardiac surgery as a complex undertaking requiring consideration of operational and implementation factors in a cascade of events whereby improved health outcomes can be realised [33,73]. The protocol incorporates relevant contemporary principles for effective screening program implementations such as inclusive recruitment, testing processes, information access, diagnosis, referral, intervention, follow-up, patient education and support, staff training and program management and evaluation [33]. Furthermore, to realize health outcome benefits, routine screening in cardiac surgery will likely create an increased demand on psychosocial health services [34,74], therefore a considered and calibrated response to screening dependent on current contextualised evidence should be an integral part of any future implementation. The EMBRACE study protocol will generate evidence and knowledge products as part of the Knowledge to Action framework to inform such a response and overall, this research will further a generalisable understanding of useful formative research activities to improve the psychosocial management of cardiac surgery patients. Using self-reported instruments for psychological case detection while not including psychiatric diagnostic interviews or physiological markers reflects the pragmatic and formative nature of the research and is balanced with the linkage of objective medical risk and outcomes data. The selected instruments are well-validated for screening purposes, reflect the patient subjective experience, which is crucial in diagnosis and treatment, and are appropriate for identifying patients who may benefit from further support without imposing additional burden or complexity in the preoperative environment.

Potential limitations of the study include a risk that the broad inclusion of surgical cases supporting real-world research translation, may lead to heterogeneity resulting in a loss of power to accommodate confounding for target psychosocial exposures, which will be considered in the analysis.

Participant sampling may be potentially biased through several mechanisms. For example, participants are drawn from a single institution, however this is the largest public cardiac surgical unit in Australia (undertaking >40% of Queensland’s public cases), providing a diverse cross-section of patients drawn across the large state, ranging from remote Indigenous centres to metropolitan localities. Future implementation research will test the developed screening model of care across broader contexts.

Decisions by the clinical care team to exclude patients from participation may be influenced by their perceptions that the study’s focus on emotional well-being could negatively impact a patient’s mental state in cases of emotional distress, such as visible upset or crying. This risks exclusion of participants who may be more likely to have positive target psychosocial variable scores but affects a very small proportion of the cohort. Nevertheless, deference to clinicians’ perceptions due to the critical nature of the cardiac surgery procedure is necessary for continued engagement with the care team and ethically necessary to minimize risk, consistent with principles of beneficence and respect [48]. While this may slightly limit generalizability, it ensures patient welfare in a study that does not offer any direct clinical benefit. Furthermore, such patients may be more likely to decline given the informed consent advice that the surveys could be triggering for some and some sub-groups may be under-represented due to fear, suspicion and stigma associated with emotional or mental health research [75]. This is an issue present in much psychosocial research and this study will specifically investigate potential for bias by comparison of the consented participant characteristics against the total cardiac surgery population characteristics including administrative coding of mental health co-morbidities and psychological Allied Health referrals and also in comparison with the aggregate characteristics of those patients not invited or declining for mentioning emotional reasons. This will add valuable insight into possible reasons for variation observed across studies for incidence and effect size of psychosocial co-morbidities not elsewhere described.

Implementation science and a pragmatic formative research approach emphasises translating evidence into practice, necessarily addressing real world questions and contextualising evidence, which in turn informs program planning [42] and theory for how and why the proposed psychosocial screening model of care will work. While the external validity of this study’s findings should be carefully judged, the EMBRACE protocol as a process for developing and delivering a successful screening program should be widely applicable. It is anticipated that a future co-designed screening model of care will be tested in more diverse centres and contexts as part of an Implementation Science process.

## Dissemination

The findings of this study will be disseminated through publication in the peer-reviewed international health and medical scientific literature and the presentation of research at local, national and international conferences. Additionally, communication opportunities aimed at patients and the general public will be sought at various forums and publication avenues.

## Supporting information

S1 Checklist EQUATOR reporting checklist: Guidance for reporting intervention development studies in health research (GUIDED). (DOCX)

S1 Variables Clinical variables and definitions according to QCOR Collection form. (PDF)

S1 Letter Patient invitation letter. (PDF)

S1 PICF Participant information and consent form. (PDF)

S1 Script Interview guide script. (PDF)
