# Correction: Formative research for a pre-operative psychosocial screening program for cardiac surgical patients: The EMBRACE study, a mixed methods knowledge to action protocol

**DOI:** 10.1371/journal.pone.0348211

**Published:** 2026-04-24

**Authors:** Susan E. Smith, Marlien Varnfield, David J. Kavanagh, Tricia Rolls, Usha Gurunathan, Bo Janoschka, Karen Hay, Rishendran Naidoo, Jed Duff, Esben Strodl

In Fig 1, the last three questions from the survey form were omitted. Please see the correct Fig 1 here.

**Fig 1:**
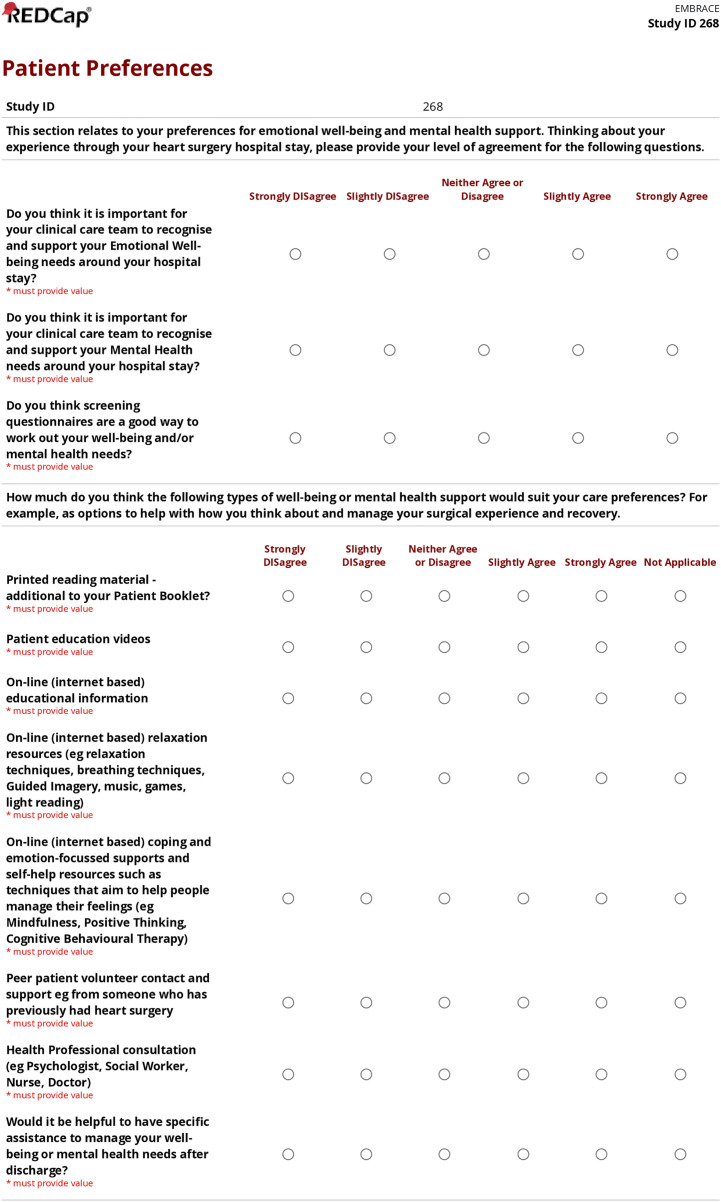
Patient preferences for psychosocial supports survey.
