# Supplementary material for: Formative research for a pre-operative psychosocial screening program for cardiac surgical patients: The EMBRACE study, a mixed methods knowledge to action protocol
Source: PLoS One. 2025 Dec 30;20(12):e0322592. doi: 10.1371/journal.pone.0322592 (PMC12752988; doi:10.1371/journal.pone.0322592)
Supplement: S1 Variables — (PDF) [file pone.0322592.s002.pdf]

Statewide Cardiac Clinical Informatics Unit
Cardiac Surgery Data Collection Form
Contact: SCCIU@health.qld.gov.au

Statewide Cardiac Clinical Informatics Unit

Cardiac Surgery Data Collection Form

CARDIAC SURGERY DATA COLLECTION FORM

Facility:

URN:

Family name:

First name:

Middle name:

Date of birth: / /

Sex: ○M ○F ○I

Patient details

**Address**	
**Suburb**	
**State**	○QLD ○NSW ○VIC ○SA ○NT ○ACT ○WA ○TAS *OR* ○Overseas ○Unknown
**Post code**	
**Phone number 1**	
**Phone number 2**	
**Email**	
**Medicare number**	*OR* □Not registered
**Aboriginal or Torres Strait Islander?**	○Yes ○No *If Yes* **Aboriginal** ○Yes ○No; **Torres Strait Islander** ○Yes ○No

Admission

**Admission date**	/ /
**Date transferred from CTS care**	/ /
**Discharge date**	/ /
**Insurance**	○Private ○Medicare ○Self-insured ○Overseas ○Other ○DVA* **If DVA* **DVA file number**
**Elective day of surgery/DOSA**	○Yes ○No

Operation

**Surgery date**	/ /
**Operation number of the day for this patient**	○1 ○2 ○3 ○4 ○5 ○6 ○Unobtainable ○Pending
**Comment**	

V3.0 – Jan 2019

<table>
<tr><th>Risk factors</th><th></th></tr>
<tr><td>Current smoker</td><td>Has the patient used tobacco products within one month of the index operation? Note: Tobacco products include (but are not limited to) cigarettes, cigars, pipes, and chewing tobacco.</td></tr>
<tr><td>Diabetes</td><td>Does the patient have a history of diabetes (regardless of the duration of disease or need for anti- diabetic agents)?</td></tr>
<tr><td>Diabetes Control</td><td>Indicate the most aggressive diabetes control therapy at the time of surgery.
Note: Aggressiveness of treatment is ranked in the order: insulin > oral > diet</td></tr>
<tr><td>Hypercholesterolaemia</td><td>Does the patient have a history of hypercholesterolaemia diagnosed and/or treated by a physician, and/or Cholesterol > 5.0mmol/L, HDL <1.0mmol/L or Triglycerides >2.0mmol/L?</td></tr>
<tr><td>Last pre-op creatinine level</td><td>Record the patients last serum creatinine level recorded prior to the index operation</td></tr>
<tr><td>Dialysis</td><td>Is the patient on pre-operative dialysis?
Note: Renal dialysis includes Haemodialysis (HD), Peritoneal Dialysis (PD) and Acute Renal Replacement Therapy (ARRT) which includes Haemofiltration and Haemodiafiltration</td></tr>
<tr><td>Hypertension</td><td>Does the patient have a diagnosis of hypertension, documented by any one or more of the following?
a. Documented history of hypertension diagnosed and treated with medication, diet, and/or exercise.
b. Blood pressure > 140 systolic or >90 diastolic on at least two occasions.
c. Current use of antihypertensive medication.</td></tr>
<tr><td>Cerebrovascular disease</td><td>Does the patient have a history of cerebrovascular disease (CBVD), documented by any of the following?
a. Unresponsive coma >24 hours at any time prior to the index admission.
b. Cerebrovascular accident (CVA) with symptoms remaining > 72 hours after onset.
c. Reversible ischaemic neurologic deficit (RIND) with recovery within 72 hours.
d. Transient ischaemic attack (TIA) with recovery within 24 hours.
e. e. Non-invasive carotid test with 50% diameter stenosis (equivalent to 75% cross-sectional area stenosis).</td></tr>
<tr><td>Cerebrovascular disease Type</td><td>Indicate the most severe type of cerebrovascular disease (CVD) the patient has from the list.
Note: Severity of CVD is ranked in the following order: Coma > CVA > RIND/TIA > Carotid
1. Unresponsive coma > 24 hours at any time prior to the index admission: Patient experienced complete cerebral unresponsiveness with no evidence of appropriate psychological or physiological responses to stimulation.
2. CVA: Patient has a history of stroke characterised by loss of neurological function with residual symptoms > 72 hours after onset.
3. RIND/TIA:
a. RIND – Patient has experienced loss of neurological function with symptoms remaining > 24 hours after onset but with complete return of function within 72 hours OR:
b. TIA – Patient has experienced loss of neurological function that was abrupt in onset but with complete return of function within 24 hours.
4. Carotid test: Non-invasive/invasive carotid test demonstrating 50% or greater diameter stenosis (equivalent to 75% cross-sectional area stenosis).</td></tr>
<tr><td>Carotid test result</td><td>Does the result of a non-invasive/invasive carotid test indicate 50% or greater diameter stenosis (equivalent to 75% cross-sectional area stenosis)?</td></tr>
<tr><td>Peripheral vascular disease (PVD)</td><td>Does the patient have a history of PVD characterised by aneurysmal, chronic, or acute occlusion/narrowing of the arterial lumen of the aorta or extremities? Examples include:
a. Claudication either with exertion or rest.
b. Amputation for arterial insufficiency.
c. Vascular reconstruction, bypass surgery, or percutaneous intervention to the extremities.
d. Documented aortic aneurysm.
e. Documented renal artery stenosis.
f. Positive non-invasive testing documented (e.g. ankle brachial index < 0.8).</td></tr>
<tr><td>Infective endocarditis - Type</td><td>Indicate if endocarditis is active or treated.
1. Active: The patient is currently on antibiotic therapy for endocarditis.
2. Treated: No antibiotic medication (other than prophylactic medication) is being given at the time of surgery.</td></tr>
<tr><td>Organism</td><td>Indicate the organism responsible for the infective endocarditis.
Note: Cultures obtained in the operating theatre may be used.
<table>
<tr><td>Abiotropihia defectiva</td><td></td><td>Aggregatibacter</td><td></td><td>Candida albicans</td><td></td></tr>
<tr><td>Candida dubliniensis</td><td></td><td>Candida parapsilosis</td><td></td><td>Enterococcus faecalis</td><td></td></tr>
<tr><td>Enterococcus gallinarus</td><td></td><td>Granulicatella adiacens</td><td></td><td>Granulicatella elegans</td><td></td></tr>
<tr><td>Group G Streptococcus bacteraemia</td><td></td><td>MRSA</td><td></td><td>MSSA</td><td></td></tr>
<tr><td>Haemophilus parainfluenzae</td><td></td><td>Propionibacterium acnes</td><td></td><td>Pseudomonas aeruginosa</td><td></td></tr>
<tr><td>Rothia dentocariosa</td><td></td><td>Staphylococcus aureus</td><td></td><td>Staphylococcus epidermidis</td><td></td></tr>
<tr><td>Staphylococcus lugdunensis</td><td></td><td>Streptococcus agalactiae</td><td></td><td>Streptococcus anginosus</td><td></td></tr>
<tr><td>Streptococcus gordonii</td><td></td><td>Streptococcus mitis</td><td></td><td>Streptococcus mutans</td><td></td></tr>
<tr><td>Streptococcus salivarius</td><td></td><td>Streptococcus sanguinis</td><td></td><td>Organism unidentified</td><td></td></tr>
<tr><td>No organism identified</td><td></td><td>Other</td><td></td><td></td><td></td></tr>
</table></td></tr>
<tr><td>IVDU History</td><td>Please describe IVDU history:
1. Current: 0 – 3 Month since last injection
2. Previous: > 3 months since last injection</td></tr>
<tr><td>Respiratory disease</td><td>Does the patient have a chronic respiratory/lung disease which is characterised by any of following?
1. Mild Patient is on chronic inhaled or oral bronchodilator therapy.
2. Moderate Patient is on chronic oral steroid therapy directed at lung disease.
3. Severe p02 on room air < 60 or pCO2 on room air > 50 or mechanical ventilation for chronic lung disease.</td></tr>
<tr><td>Pulmonary hypertension</td><td>Does the patient have pulmonary hypertension as defined by the highest pulmonary artery systolic pressure (PASP) measured via right heart catheterisation or Echocardiography. Please specify severity:
1. Mild: PASP = 20-30mmHg
2. Moderate: PASP = 31-55mmHg
3. Severe: PASP >55mmHg</td></tr>
<tr><td>Immunosuppressive therapy</td><td>Was the patient on any form of immunosuppressive therapy within 30 days of the current surgery, or is the patient on any steroids for chronic long-term use? This includes, but is not limited to systemic steroid therapy equivalent to ≥5mg prednisolone within 30 days, anti-rejection medication and chemotherapy.
Note: Drug classes that are considered to be immunosuppressive include:
- Corticosteroids (only if taken systemically)
- Cytotoxic drugs
- Antimetabolites (cyclosporine)
- Monoclonal antibodies</td></tr>
<tr><td>Preoperative haemoglobin</td><td>Record the patient's last haemoglobin recorded prior to surgery.</td></tr>
</table>

Risk factors

**Smoking history**	○Yes ○No	*If Yes*	**Current smoker**	○Yes ○No ○Unknown
**Diabetes**	○Yes ○No	*If Yes*	**Control**	○None ○Diet ○Oral ○Insulin
**Poor mobility**	○Yes ○No	*Select "Yes" where there is severe impairment of mobility secondary to musculoskeletal or neurological dysfunction*		
**Hypercholesterolaemia**	○Yes ○No	*If Yes*	**Statin therapy prior to presentation**	○Yes ○No
**Last pre-op creatinine level**	☐☐☐☐ µmol/L			
**Dialysis**	○Yes ○No			
**Transplant**	○Yes ○No			
**Hypertension**	○Yes ○No			
**Risk scores**				
**Logistic EuroSCORE**	☐☐ %			
**Parsonett**	☐☐			
**Cerebrovascular disease**	○Yes ○No	*If Yes*	**Type**	○Coma<br>○CVA*<br>○RIND or TIA<br>○Carotid test
		**If CVA*	**When**	○CVA ≤2wks ○CVA >2wks
**Carotid test result**	○Yes ○No			
**Peripheral vascular disease**	○Yes ○No			
**Infective endocarditis**	○Yes ○No	*If Yes*	**Type**	○Active*<br>○Treated
**If Active* **Site:**	○Valve ○Other	*If Other*	**Specify**	□ Aortic root<br>□ Mitral annulus<br>□ Intracardiac shunt<br>□ Prosthetic valve<br>□ Pacemaker
**Organism (specify)**	☐	*OR*		○Organism unidentified<br>○No organism identified
**History of IV drug use**	○Yes ○No ○Unknown	*If Yes*	**Most recent**	○0-3 months<br>○>3 months
**Respiratory disease**	○Yes ○No	*If Yes*	**Type**	○Mild<br>○Moderate<br>○Severe
**Preoperative COVID-19 diagnosis**	○Yes ○No			
**Pulmonary hypertension**	○Yes ○No	*If Yes*	**Type**	○Mild (PA systolic 20-30mmHg)<br>○Moderate (PA systolic 31-55 mmHg)<br>○Severe (PA systolic >55 mmHg)
**Immunosuppressive therapy**	○Yes ○No			
**Preoperative haemoglobin**	☐☐☐ (40 to 200g/L)			

Risk factors ctd.	
Previous MI - Type	**Non ST-Elevation Myocardial Infarction (NSTEMI)** – AT LEAST ONE of the following biomarkers for detecting myocardial necrosis MUST be present: (refer to Note regarding Reference Control Limits):<br>1. **<u>Troponin T or I</u>:** Maximal concentration of troponin T or I > the MI diagnostic limit on at least one occasion within the first 24 hours from the index clinical event;<br>2. **<u>CK-MB:</u>**<br>• Maximal value of CK-MB > 2 x the upper limit of normal (ULN) on one occasion during the first hours after the index clinical event; OR<br>• Maximal value of CK-MB (preferable CK-MB mass) > ULN on two successive samples.<br>3. **<u>Total CK</u>**: Only where Troponin or CK-MB assays are unavailable, total CK > 2 x the ULN (or the B fraction of CK) may be employed.<br>**Note:** The preferred assays to use as biomarkers for myocardial necrosis are Troponin, CK-MB or total CK (in that order).<br>**AND ONE** of the following:<br>1. ST segment depression or T wave abnormalities in the ECG; OR<br>2. In the presence or absence of chest discomfort. Ischemic symptoms may include:<br>• Unexplained nausea and vomiting; or<br>• Persistent shortness of breath secondary to left ventricular failure; or<br>• Unexplained weakness, dizziness, light- headedness, or syncope.<br><br>**ST-Elevation Myocardial Infarction (STEMI)** – AT LEAST ONE of the following biochemical indicators for detecting myocardial necrosis MUST be present (refer to Note regarding Reference Control Limits):<br>1. **<u>Troponin T or I</u>:** Maximal concentration of troponin T or I > the MI diagnostic limit on at least one occasion within the first 24 hours from the index clinical event;<br>2. **<u>CK-MB</u>:**<br>• Maximal value of CK-MB > 2 x the upper limit of normal (ULN) on one occasion during the first hours after the index clinical event; OR<br>• Maximal value of CK-MB, (preferable CK-MB mass) > ULN on two successive samples.<br>3. **<u>Total CK</u>:** Only where Troponin or CK-MB assays are unavailable, total CK > 2 x the ULN (or the B fraction of CK) may be employed.<br>Note: The preferred assays to use as biomarkers for myocardial necrosis are Troponin, CK-MB or total CK (in that order).<br>AND ONE of the following ECG changes:<br>1. **<u>ST-segment elevation</u>:** New or presumed new ST segment elevation at the J point in two or more contiguous leads with the cut-off points >=0.2 mV in leads V1, V2, or V3, or ≥0.1 mV in other leads;<br>2. **<u>Development of any Q wave</u>** in leads V1 through V3, or the development of a Q-wave ≥ 30 ms (0.03s) in leads I, II, aVL, aVF, V4, V5, or V6. (Q wave changes must be present in any two contiguous leads, and be ≥ 1mm in depth.)<br><br>**Defining Reference Control Values (MI Diagnostic Limit and Upper Limit of Normal):** Reference values must be determined in each laboratory by studies using specific assays with appropriate quality control, as reported in peer-reviewed journals. Acceptable imprecision (coefficient of variation) at the 99th percentile for each assay should be defined as < or = to 10%. Each individual laboratory should confirm the range of reference values in their specific setting
CCS class	Indicate the patient's highest Canadian Cardiovascular Society (CCS) classification leading to the current episode of hospitalisation and/or intervention.<br>1. No angina symptoms.<br>2. Ordinary physical activity, such as walking or climbing the stairs does not cause angina. Angina may occur with strenuous, rapid or prolonged exertion at work or recreation.<br>3. There is slight limitation of ordinary activity. Angina may occur with moderate activity such as walking or climbing stairs rapidly, walking uphill, walking or stair climbing after meals or in the cold, in the wind, or under emotional stress, or walking more than two blocks on the level, and climbing more than one flight of stairs at a normal pace under normal conditions.<br>4. There is marked limitation of ordinary physical activity. Angina may occur after walking one or two blocks on the level or climbing one flight of stairs under normal conditions at a normal pace.<br>5. There is inability to carry on any physical activity without discomfort; angina may be present at rest.
IV GTN	Did the patient receive IV GTN to treat angina on the day of surgery?
IV heparin	Did the patient receive IV Heparin to treat angina ≤12 hours prior to surgery?
Full dose heparinoids	Did the patient receive full dose Heparinoids to treat angina **≤24 hours prior to surgery?**<br><br>Examples of treatment include subcutaneous Clexane (Enoxaparin) at ≥1mg/kg bi-daily. Treatment also includes use of other low molecular weight heparinoids (LMWH) at an equivalent dose. Examples of LMWH include but are not limited to Fragmin (Dalteparin) and Clexane (Enoxaparin)
History of congestive heart failure (CHF)	Does the patient have a history of congestive heart failure (CHF) diagnosed by a physician and evidenced by **TWO** of the following?<br>a. Paroxysmal nocturnal dyspnoea (PND) or orthopnoea.<br>b. Shortness of breath on exertion (SOBOE) due to heart failure i.e. not as an angina equivalent.<br>c. Chest x-ray (CXR) showing pulmonary congestion.<br>d. Patient received treatment for CHF by use of ACE inhibition, diuretics, Carvedilol (Coreg).
CHF at current admission	**During** the current admission, was the patient diagnosed with CHF or was the management of CHF changed due to deterioration in CHF?
NYHA class	Indicate the patient's highest level of dyspnoea as classified by the New York Heart Association (NYHA) leading to the current episode of hospitalisation and/or procedure.<br>**Note**: Dyspnoea in a patient who has only coronary artery disease (CAD) should be considered an angina equivalent and therefore indicated by CCS class. The NYHA class should be marked 1 in such patients.<br>**I.** Patient with cardiac disease but without resulting limitation of physical activity. Ordinary physical activity does not cause undue fatigue, palpitations or dyspnoea.<br>**II.** Patients with cardiac disease resulting in slight limitation of physical activity. They are comfortable at rest. Ordinary physical activity results in fatigue, palpitations or dyspnoea.<br>**III.** Patients with cardiac disease resulting in marked limitation of physical activity. They are comfortable at rest. Less than ordinary physical activity results in fatigue, palpitations, or dyspnoea.<br>**IV.** Patients with cardiac disease resulting in inability to carry on any physical activity without discomfort. Symptoms of cardiac insufficiency may be present even at rest. If any physical activity is undertaken, discomfort is increased.
Cardiogenic shock	Was the patient in cardiogenic shock at the time of procedure? Only code yes if all of the following criteria apply:<br>1. Sustained (>30 minutes) episode of systolic blood pressure <90mmHg or the requirement for parenteral inotropic or vasopressor agents or mechanical support (e.g. Intra-aortic balloon pump (IABP), extracorporeal circulation, ventricular assist devices to maintain BP >90mmHg); **AND**<br>2. Evidence of elevated filling pressures (e.g. elevated PAWP or pulmonary oedema on examination or chest radiograph); **AND**<br>3. Evidence of end organ hypoperfusion (e.g. urine output <30mL/hour; or cold/diaphoretic extremities; or altered mental status, etc.)

Preoperative status

**Previous myocardial infarction (MI)**	○Yes ○No	*If Yes*	**Type**	○NSTEMI ○STEMI ○Unknown
			**When**	○≤6hrs ○>6hrs and <24hrs<br>○1-7 days ○8-21 days<br>○22-90 days ○>90 days
**CCS class**	○0 ○1 ○2 ○3 ○4	*If > 0*	**IV GTN**	○Yes ○No
			**IV Heparin**	○Yes ○No
			**Full dose heparinoids**	○Yes ○No
**History of congestive heart failure (CHF)**	○Yes ○No	*If Yes*	**CHF at current admission**	○Yes ○No
**NYHA class**	○I ○II ○III ○IV			
**Cardiogenic shock**	○Yes ○No			
**Resuscitation (within one hour pre-op)**	○Yes ○No			
**Arrhythmia**	○Yes ○No	*If Yes*	**Atrial**	○Yes* ○No
			*If Yes **Type**	○Paroxysmal<br>○Permanent<br>○Unknown
			**Heart block**	○Yes ○No
			**Ventricular**	○Yes ○No
			**Other**	○Yes ○No
**Permament pacemaker in situ**	○Yes ○No			

Medications at time of surgery

**Inotropes**	○Yes ○No	*If Yes*	**Specify**	
**IV nitrates**	○Yes ○No			
**Anticoagulation therapy**	○Yes ○No			
**Steroids**	○Yes ○No			

Aspirin or other antiplatelet therapy within 7 days of surgery

**Aspirin**	○Yes ○No	*If Yes*	**When (days)**	○<1 ○1 ○2 ○3 ○4 ○5 ○6 ○7
**Thienopyridine**	○Yes ○No	*If Yes*	**When (days)**	○<1 ○1 ○2 ○3 ○4 ○5 ○6 ○7
**Ticagrelor**	○Yes ○No	*If Yes*	**When (days)**	○<1 ○1 ○2 ○3 ○4 ○5 ○6 ○7
**Tyrofiban or Eptifibatide**	○Yes ○No	*If Yes*	**When (days)**	○<1 ○1 ○2 ○3 ○4 ○5 ○6 ○7
**Abciximab**	○Yes ○No	*If Yes*	**When (days)**	○<1 ○1 ○2 ○3 ○4 ○5 ○6 ○7
**Other antiplatelet**	○Yes ○No	*If Yes*	**When (days)**	○<1 ○1 ○2 ○3 ○4 ○5 ○6 ○7

**Critical preoperative status**	○Yes ○No	*Select "Yes" if any of the following apply:*<br>*- Ventricular tachycardia* *- Ventricular fibrillation*<br>*- Aborted sudden death* *- Preoperative cardiac massage*<br>*- Preoperative ventilation before anaesthetic room*<br>*- Preoperative inotropes or IABP*<br>*- Preoperative acute renal failure (anuria or oliguria <10ml/hr)*

Previous Interventions	
Previous cardiothoracic intervention – open or percutaneous	Does the patient have any history of previous cardiothoracic intervention **(surgical or percutaneous)** prior to the index operation (including prior procedures performed in the current admission)?<br>***Note: Code 'yes' for all forms of percutaneous angioplasty and transcatheter cardiac interventions INCLUDING prior interventions in the same admission episode e.g. the patient had a prior PTCA/stent at another hospital before transfer to current hospital for surgery.***
Requiring cardiopulmonary bypass	Record the number of cardiac surgical operations performed on this patient utilising cardiopulmonary bypass ***prior to the index operation***.
Without cardiopulmonary bypass (beating heart surgery)	Record the number of cardiac surgical operations performed on this patient without cardiopulmonary bypass ***prior to the index operation.***
CABG	Has the patient undergone a previous coronary artery bypass surgery through any approach prior to the index operation (including those performed in the current admission)?
Off-pump CABG	Was the previous coronary artery bypass graft surgery performed without the use of cardiopulmonary bypass through any approach?
TAVR	Was a transcatheter aortic valve replacement (TAVR) procedure performed at any time prior to the index operation (including those performed in the current admission)?
TMVR	Was a transcatheter mitral valve replacement (TMVR) procedure performed at any time prior to the index operation (including those performed in the current admission)?
PTCA/stent	Was a transluminal coronary angioplasty/coronary atherectomy and/or coronary stent procedure performed at any time prior to the index operation (including prior procedures performed in the current admission)?
In which admission?	Was a PTCA/stent procedure performed prior to the current admission (remote) or during the current admission (this admission)?<br>***Note: Include patients that were directly transferred from the hospital where the percutaneous intervention procedure was performed to the current hospital where index operation will be performed as this admission.***
Interval	If PTCA/Stent was performed in this admission:<br>Record the time interval between PTCA/atherectomy/stent and the current surgical procedure. The time interval should be recorded as the number of hours that have lapsed since previous PTCA/ atherectomy /stent and the current surgical procedure.<br>***Note: Includes patients that were directly transferred from the hospital where the percutaneous intervention procedure was performed to the current hospital where index operation will be performed.***
Non-surgical balloon valvuloplasty	Has the patient undergone a non-surgical balloon valvuloplasty at any time prior to the index operation (including those done in the current admission)?
ASD device closure	Has the patient undergone a percutaneous procedure for closure of an atrial septal defect or patent foramen ovale prior to the index operation (including those in the current admission)?
VSD device closure	Has the patient undergone a percutaneous procedure for closure of a ventricular septal defect prior to the index operation (including those in the current admission)?
Left atrial appendage occlusion	Has the patient undergone occlusion of the atrial appendage prior to the index operation (including those in the current admission) by either of the following techniques:<br>1. **Intracardiac** – e.g. Watchman or Amplatzer OR<br>2. **Extracardiac** – e.g. Thoracoscopic insertion of clips.
Percutaneous SVT/VT ablation	Has the patient undergone a percutaneous ablation procedure for any form of supraventricular tachycardia (SVT) or ventricular tachycardia prior to the index operation (including those done in the current admission)?
Percutaneous mitral valve repair	Has the patient undergone percutaneous mitral valve repair (e.g. Mitraclip) prior to the index operation (including those done in the current admission)?

Previous cardiothoracic intervention – open or percutaneous

**Previous cardiac surgery**	○Yes ○No	*If Yes*	**Requiring cardiopulmonary bypass**	○0 ○1 ○2 ○3 ○4 ○5 ○6 ○7 ○8 ○9
			**Without cardiopulmonary bypass (beating heart surgery)**	○0 ○1 ○2 ○3 ○4 ○5 ○6 ○7 ○8 ○9
			**CABG**	○Yes ○No
			**Off-pump CABG**	○Yes ○No
			**Valve**	○Yes ○No
			**Other cardiac**	○Yes ○No
**Previous percutaneous intervention**	○Yes ○No	*If Yes*	**TAVR**	○Yes ○No
			**TMVR**	○Yes ○No
			**PTCA/stent**	○Yes* ○No
			*If Yes — **Which admission**	○This admission** ○Remote
			**If this admission — **Interval**	☐☐☐ hours
			**Non surgical balloon valvuloplasty**	○Yes ○No
			**ASD device closure**	○Yes ○No
			**VSD device closure**	○Yes ○No
			**Left atrial appendage occlusion**	○Yes ○No
			**Percutaneous SVT/VT ablation**	○Yes ○No
			**Percutaneous mitral valve repair**	○Yes ○No

Previous congenital surgery ○Yes ○No *If Yes:*

- □ Closed valvotomy
- □ Atrial septectomy
- □ Repair of atrial septal defect (secundum)
- □ Patent ductus arteriosus closure or pulmonary artery band
- □ Systemic to pulmonary artery shunt
- □ Thoracic vessel repair, direct
- □ Open valvotomy
- □ Annuloplasty
- □ Infundibulectomy
- □ Subaortic stenosis resection
- □ Repair of ventricular septal defect
- □ Repair of endocardial cushion defect (partial or complete AV canal)
- □ Repair of Tetralogy of Fallot
- □ Thoracic vessel repair, prosthesis
- □ Septal surgery, unspecified
- □ Operation adjacent to heart valves – supravalve stenosis
- □ Revision of procedure
- □ Valve replacement
- □ Repair of total anomalous pulmonary venous connection
- □ Repair of truncus arteriosus
- □ Ebsteins
- □ Right ventricular-pulmonary artery conduit
- □ Left ventricular-aorta conduit
- □ Cavo-pulmonary shunt – Hemifontan/Glenn
- □ Repair of vessel, including aorto-pulmonary window
- □ Pulmonary atresia
- □ Arterial switch
- □ Total cavo-pulmonary anastomosis – Fontan
- □ Diagnosis of hypoplastic left heart syndrome - Norwood
- □ Other* (Specify below)

***Specify:** ____________________

**Heamodynamics**	
Cardiac catheterization (angiogram or pressure study)	Has the patient had cardiac catheterisation (angiography)?
Date of cardiac catheterization	Record the date of the most recent cardiac catheterisation.<br>Enter **09/09/9999** for unknown date.
LVEF method	Did the patent have their left ventricular ejection fraction (LVEF) measured? Indicate the method was used.<br>• Not measured. **OR**<br>• Angiography – LV gram during cardiac catheterisation. **OR;**<br>• Radionuclide – nuclear medicine MPS **OR;**<br>• Echocardiography: transthoracic or transoesophageal echocardiography. **OR;**<br>• Magnetic Resonance Imaging.
Ejection fraction (EF)	Record the percentage of blood emptied from the left ventricle at the end of the contraction. Use the most recent determination prior to intervention.
Left main stenosis > 50%	Does the patient have any stenosis that involves any part of the left main? Left main coronary stenosis is present when there is >50% compromise of vessel diameter in any angiographic view.
Number diseased coronary systems	Indicate the number of (the three) major coronary systems (LAD system, Circumflex system, Right Coronary system) with >50% narrowing in any angiographic view. The number of diseased systems should be the number of systems requiring surgical approach at that operation.<br>**Note:**<br>• **Left Main Disease (>50%) is counted as TWO systems (LAD and Circumflex). Left main and RCA would count as THREE in total and dominant circumflex would count as TWO systems.**<br>• **LMCAD associated with dominant Circumflex counts as THREE systems.**<br><br>**If a system has been grafted previously and the graft has no haemodynamically significant stenosis, then that system is NOT counted as diseased. If a previous graft requires replacement, then that system IS counted as diseased.**<br><br>**ALWAYS ANSWER THIS QUESTION. IF THERE ARE NO DISEASED CORONARY ARTERY SYSTEMS THEN INDICATE 0.**
**Operation status**	
Status	Indicate the clinical status of the patient prior to entering the operating room from the table below:<br>1. **Elective**: the procedure could be deferred without risk of compromised cardiac outcome.<br>2. **Urgent:** Not routine - clinical reason for operating this admission:<br>a. Within 72 hours of angiography if index operation was performed in the same admission as angiography (here ‘same admission’ includes situations where angiography was performed in another hospital prior to direct transfer to current hospital where index operation is to be performed). OR<br>b. Within 72 hours of an unplanned admission (in patients who had a previous angiogram and was scheduled for surgery but was admitted acutely). OR<br>c. Procedure required during same hospitalisation in a clinically compromised patient in order to minimise chance of further clinical deterioration.<br>3. **Emergency:** Scheduled surgery required in the next available theatre on the same day (as admission) due to refractory angina or haemodynamic compromise.<br>4. **Salvage:** The patient underwent CPR en route to, or in the operating room, prior to surgical incision.
Reason	Indicate why the patient’s clinical status was ‘urgent’:<br>1. Acute Myocardial Infarction (AMI) stabilised and not requiring emergency operation.<br>2. Pre-op Intra-Aortic Balloon Pump (IABP).<br>3. Threatening coronary anatomy with acute symptoms.<br>4. Unstable angina requiring IV therapy.<br>5. Severe acute valve dysfunction either native or prosthetic

**Haemodynamics**				
**Height**	[ ][ ][ ] cm			
**Weight**	[ ][ ][ ] kg			
**Cardiac catheterization (angiogram/pressure study)**	○Yes ○No	*If Yes*	**Date**	[ ] / [ ] / [ ]
**LVEF method**	○No ○LV Gram ○Radionuclide ○Echo ○MRI ○Unknown			
**Ejection fraction (EF)**	[ ][ ] (5-90%)			
**Left main stenosis > 50%**	○Yes ○No			
**Number of diseased coronary systems**	○None ○One ○Two ○Three			

**Operation status**				
**Consultant surgeon**	[ ][ ][ ][ ] (Code)			
**Operating surgeon (proceduralist)**	○Consultant ○Senior Registrar ○Trainee<br>○Overseas Fellow ○Oversight			
**Procedure status**	○Elective<br>○Urgent<br>○Emergency<br>○Salvage	*If Urgent*	**Reason**	○AMI<br>○Pre-op IABP<br>○Coronary anatomy<br>○Unstable angina<br>○Severe acute valve dysfunction
**Direct transfer from cath lab to theatre**	○Yes ○No			
**Coronary artery bypass**	○Yes ○No	*If Yes*	→ *Complete Coronary Artery Bypass section*	
**Valve surgery**	○Yes ○No	*If Yes*	→ *Complete Valve Surgery section*	
**Cardiac transplant**	○Yes ○No	*If Yes*	**Type**	○Atrial<br>○Bicaval
			**Category**	○Primary<br>○Redo<br>○VAD Explant
**Lung transplant**	○Yes ○No	*If Yes*	**Type**	○Single lung - Whole<br>○Single lung - Lobe<br>○Double<br>○BSSLTx<br>○Other*
			**If Other* **Specify**	[ ]
**Cardiopulmonary transplant**	○Yes ○No			
**LV aneurysm**	○Yes ○No			
**VSD (acquired)**	○Yes ○No			
**ASD**	○Yes ○No			
**Trauma**	○Yes ○No			
**LVOT myectomy for HOCM**	○Yes ○No			
**LV rupture repair**	○Yes ○No			
**Pericardiectomy**	○Yes ○No			
**Pulmonary thrombo-endarterectomy**	○Yes ○No			

Operation status ctd.	
Atrial arrhythmia surgery	Is the index operation for paroxysmal, persistent or permanent atrial tachyarrhythmia?
Lesion set	Indicate the predominant lesion set from the following:<br>1. ***Cox-Maze III:*** Complete Maze III lesions as described by James Cox<br>2. **Radial:** Radiating lesions from the SA node as described by Nitta or Westmead Group.<br>3. **Mini Maze:** Circumferential pulmonary veins isolation and line of conduction block in both right and atrial isthmus and left atrial isthmus (including coronary sinus).<br>4. **Left atrial reduction:** Circumferential pulmonary veins isolation with excision or exclusion of cuff of atrial tissue to reduce left atrial dimensions.<br>**Note:** this does not include mere removal of the left atrial appendage<br>5. **Pulmonary vein isolation:** Circumferential pulmonary vein isolation only.<br>6. **Left atrial only:** Left atrial component of Cox-Maze II.<br>7. **Right atrial only**: Right atrial component of Cox-Maze II.<br>8. **Other:** When lesion set does not meet the above definitions.<br>9. **Cox-MAZE IV:** A procedure designed to treat atrial fibrillation. It is performed on CPB, creating the same ablation lines as a Cox III, but through limited left and right atriotomies.
Aortoplasty	Is the aortic procedure a Direct Aortoplasty?<br>**Note: Defined as a resection of a segment of aorta with direct suture closure or suture plication of a segment of aorta to reduce diameter.**
Replacement	Is the aortic procedure classified as a replacement?<br>***Note: Replacement is defined as circumferential replacement of the aorta, performed for aortic pathology. It does not include incidental replacement of a short segment of the ascending aorta as part of an aortic root replacement.***
Endarterectomy	Has an extensive Endarterectomy been performed?<br>***Note: Extensive is defined by more than one quarter of the circumference of the aorta, and more than 2 cm wide.***
Patch repair	Has the aorta been repaired with a patch after resection or to enlarge it?<br>***Note: Does NOT include patch placed to the aortic root to facilitate closure after aortic valve replacement.***
Conduit replacement	Does the aortic surgery require conduit replacement?
External aortic root support	Does the aortic surgery require an external aortic root support? Sometimes called a Personalised External Aortic Root Support (PEARS) operation.
Ascending	Does the aortic replacement involve the ascending aorta?
Arch	Does the aortic replacement involve the aortic arch?
Descending	Does the aortic replacement involve the descending aorta?
Thor/abd	Does the aortic replacement involve the thoraco-abdominal aorta?
Other non-cardiac procedure	Does the index operation include a non-cardiac procedure?
Carotid endarterectomy	Does the index operation include surgical removal of stenotic atheromatous plaque(s) from the carotid artery?
Lung resection	Does the index operation include surgical removal of damaged or diseased portion(s) of the lung?
Other vascular surgery	Does the index operation include procedures correcting peripheral vascular stenosis or occlusion?<br>***Note****: A peripheral vascular procedure may include procedures such as femoral artery repair, iliac artery repair etc*
Other thoracic surgery	Does the index operation include procedure involving the thorax/pleura?
Other	Does the index operation include any other concomitant surgery not specified in the non-cardiac procedures listed?
Reason	If the index operation is classified as any type of concomitant surgery no listed above, record the specific procedure performed.

Operation status (continued from page 5)					
**Left ventricular reconstruction**	○Yes	○No			
**Pulmonary embolectomy**	○Yes	○No			
**Cardiac tumour**	○Yes	○No			
**Other congenital**	○Yes	○No			
**Permanent LV epicardial lead**	○Yes	○No			
**Left atrial appendage closure**	○Yes	○No			
**Atrial arrhythmia surgery**	○Yes	○No	*If Yes*	**Lesion set**	○Cox-Maze III<br>○Radial<br>○Mini-Maze<br>○Left Atrial Reduction<br>○Pulmonary Vein Isolation<br>○Left Atrial Only<br>○Right Atrial Only<br>○Other<br>○Cox-Maze IV
				**Energy source**	○Cut and Sew<br>○Radiofrequency<br>○Bipolar RF<br>○Cryoblation<br>○Microwave<br>○Laser<br>○Ultrasound<br>○Other
**Any other cardiac surgery - not specified above**	○Yes	○No	If Yes	**Specify**	
**Aortic procedure**	○Yes	○No	*If Yes*	**Aortic pathology aetiology**	○Aortic aneurysm<br>○Aortic dissection ≤2 weeks<br>○Aortic dissection >2 weeks<br>○Traumactic transection<br>○Calcification<br>○Other
				**Aortoplasty**	○Yes* ○No
				** If Yes* **Specify**	□ Endarterectomy<br>□ Patch repair<br>□ Conduit replacement<br>□ External aortic root support
				**Replacement**	○Yes** ○No
				***If Yes* **Specify**	□ Ascending<br>□ Arch<br>□ Descending<br>□ Thor/abd
**Other non cardiac procedure**	○Yes	○No	*If Yes*	**Carotid endarterectomy**	○Yes ○No
				**Lung resection**	○Yes ○No
				**Other vascular surgery**	○Yes ○No
				**Other thoracic surgery**	○Yes ○No
				**Other reason (specify)**	

Minimally invasive technique	
Minimally invasive technique attempted	Was a non-standard (sternotomy or thoracotomy) incision used for the cardiac procedure (on- or off- pump)?
Performed off pump	Was the index operation performed without the use of cardiopulmonary bypass (i.e. the operation NOT performed on-pump)?
Robotically assisted	Was the procedure performed with the assistance of a robot (e.g. Da Vinci, AESOP)?

CBP and Support	
Cumulative cross clamp time	Where the index operation was performed on-pump, indicate the total number of minutes the aorta was completely cross-clamped and the heart was ischaemic during bypass.
Cumulative DHCA time	Indicate the total number of minutes patient was under deep hypothermic circulatory arrest.
Cumulative cardiopulmonary bypass time (perfusion time)	Where the index operation was performed on-pump, indicate the total number of minutes the patient was on cardiopulmonary bypass.
Haemoglobin	What was the ***lowest*** haemoglobin recorded whilst the patient was on cardiopulmonary bypass?
IABP	Did the patient have an Intra-Aortic Balloon Pump (IABP) inserted at any point during the current admission?<br>***Note: This includes pre, intra & post-operatively.***
When	Where an IABP was inserted, indicate time of earliest insertion from the list below: Select ONE of the following:<br>1. Pre-operatively – before patient enters the operating theatre.<br>2. Intra-operatively – whilst the patient is in the operating theatre<br>3. Post-operatively – after the patient leaves the operating theatre.
Indication	What was the **PRIMARY** reason for insertion of the IABP? Select ONE of the following:<br>1. Haemodynamic instability (hypotension/shock)<br>2. PTCA/PCI Support<br>3. Angina<br>4. CPB wean: Cardiopulmonary bypass (CPB) weaning failure (select this option if IABP was inserted to facilitate weaning from CPB even though the problem is haemodynamic instability).<br>5. Prophylactic
ECMO	Was the patient placed on Extra-Corporeal Membrane Oxygenation (ECMO) at any time during the current admission?<br>***Note: ECMO includes peripheral or central and Veno-Arterial or Veno-venous.***
When	Indicate the earliest use of ECMO in the current admission. Select ONE of the following:<br>1. **Pre-operatively** – before patient enters the operating theatre.<br>2. **Intra-operatively** – whilst the patient is in the operating theatre<br>Post-operatively – after the patient leaves the operating theatre.
Indication	Indicate the PRIMARY reason for use of ECMO.<br>Select ONE of the following:<br>1. Cardiac failure<br>2. Respiratory failure<br>3. Hypothermia<br>4. Rescue / Salvage
VAD	Did the patient have a ventricular assist device (VAD) implanted at any point during the current admission?<br>***Note: VAD should be inserted for a true VAD indication, either temporary or otherwise.***
When	Indicate the earliest insertion of VAD during the current admission. Select **ONE** of the following:<br>1. **Pre-operatively** – before patient enters the operating theatre.<br>2. **Intra-operatively** – whilst the patient is in the operating theatre<br>3. **Post-operatively** – after the patient leaves the operating theatre
Indication	Indicate the PRIMARY reason for inserting a VAD.<br>Select **ONE** of the following:<br>1. **Bridge to Transplantation:** Includes patients who are supported with a VAD until a heart transplant is possible.<br>2. **Bridge to recovery:** Includes patients who are supported with a VAD until heart recovery is achieved.<br>3. **Destination:** Includes patients where a heart transplant is not an option. The VAD is placed for permanent life sustaining support.<br>4. **Postcardiotomy Ventricular Failure:** Includes postcardiotomy patients who receive a VAD because of failure to separate from the heart-lung machine. Postcardiotomy refers to those patients with the inability to wean from cardiopulmonary bypass secondary to left, right or biventricular failure.<br>5. **Device Malfunction:** Includes patients who are currently VAD supported and are experiencing device failure.<br>6. **End of life:** Mechanical device pump has reached functional life expectancy and requires replacement.

Minimally invasive technique				
**Minimally invasive technique attempted**	○Yes ○No			
**Performed off pump**	○Yes ○No			
**Robotically assisted**	○Yes ○No			

CPB and support				
**Cardiopulmonary bypass used**	○Yes ○No	*If Yes*	**Cardioplegia**	○Yes* ○No
			**If Yes* **Type**	○Hyperkalaemic<br>○Bretschneider HTK<br>○Del Nido Solution
			**Cumulative cross clamp time**	☐☐☐ min
			**Cumulative DHCA time**	☐☐☐ min
			**Cumulative cardiopulmonary bypass time (perfusion time)**	☐☐☐ min
			**Haemoglobin**	☐☐ g/L
**Intra-aortic balloon pump (IABP)**	○Yes ○No	*If Yes*	**When**	○Pre-Operative<br>○Intra-Operative<br>○Post-Operative
			**Indication**	○Haemodynamic<br>○PTCA<br>○Angina<br>○CPB wean<br>○Prophylactic
**Extra corporeal membrane oxygenation (ECMO)**	○Yes ○No	*If Yes*	**When**	○Pre-operative<br>○Intra-operative<br>○Post-operative
			**Indication**	○Cardiac failure<br>○Respiratory failure<br>○Hypothermia<br>○Rescue/Salvage
**Ventricular assist device (VAD)**	○Yes ○No	*If Yes*	**When**	○Pre-Operative<br>○Intra-Operative<br>○Post-Operative<br>○Unknown
			**Indication**	○Bridge to Transplantation<br>○Bridge to Recovery<br>○Destination<br>○Post Cardiotomy Ventricular Failure<br>○Device Malfunction<br>○End of Life
**Intra operative TOE**	○Yes ○No			
**Intra operative antifibrinolytic use**	○Yes<br>○No<br>○Unknown<br>○ATACAS	*If Yes*	**Type**	○Trasylol<br>○Tranexamic Acid<br>○Other<br>○Aminocaproic Acid

**Coronary artery bypass**	
Intra-operative decision to graft coronary artery	Was a decision made during the intra-operative stage to graft the coronary artery/arteries (i.e. CCAB **not proposed** before operation commenced)?

**Valve details**	
Aetiology	What was the predominant aetiology/pathology associated with the valve?<br>1. **Rheumatic:** Valve is tricuspid, typically symmetrical and has rolled edges, shortened, fibrosed leaflets. There may be commissural fusion.<br>2. **Congenital:** Spectrum of appearances: from asymmetrical, with varying degree of fusion of (typically L-R commissure) and normally disposed coronary orifices, through to classical bicuspid valve and opposing coronary orifices. May or may not be calcified.<br>3. **Aortic Idiopathic Calcific:** Senile dystrophic calcification, not having the characteristics of 1 or 2 above<br>4. **Mitral Idiopathic Calcific:** Due to calcification ONLY, with no commissural fusion, the non-calcified portions of the leaflets are normal, there is no chordal pathology, but calcium may extend into the annulus and the sub-annular myocardium.<br>5. **Ischaemic:** Regurgitation due to ischaemia (not just associated with IHD). Evidence of papillary muscle and subjacent mural infarction (acute or chronic). MR associated with IHD and dilated LV, with intact papillary muscles (e.g. anterior infarction) should NOT be considered ischaemic<br>6. **Myxomatous degeneration:** Elongated, thickened leaflets, one or more may be prolapsing. More securely determined if associated with typical myxomatous mitral valve disease. Most securely determined if confirmed by histology.<br>7. **Failed prior repair:** Self-evident (failure not due to infection).<br>8. **Prosthetic valve failure:** Structural failure of a tissue or mechanical valve.<br>9. **Peri-prosthetic leak:** Self-evident (failure not due to infection).<br>10. **Prosthetic valve thrombosis:** Leaflet, poppet or disc restriction due to (non-infective) thrombus. Includes sub-valvular pannus formation.<br>11. **Active infection:** Characterised by clinical and pathologic features of unhealed endocarditis with patients still receiving antibiotic therapy.<br>12. **Previous infection:** Previously diagnosed endocarditis that has been treated and is quiescent.<br>13. **Marfan's:** Variety of myxomatous aortic valve disease in a patient with clinical features of Marfan's syndrome.<br>14. **Annuloaortic ectasia:** Aortic regurgitation due to dilation of the aortic annulus and sino-tubular junction (possibly also the ascending aorta) with essentially normal leaflets.<br>15. **Other degenerative disease:** Generally a myxomatous valve but associated with a systemic syndrome other than Marfan's (Sjogren, Lupus, Ehlers-Danlos, etc.)<br>16. **Dissection:** Regurgitation in a previously normal valve due to dissection disrupting the commissural attachments<br>17. **Tumour:** Typical macroscopic characteristics of tumour, preferably with histological confirmation.<br>18. **Trauma:** Due to mechanical trauma.<br>19. **Iatrogenic:** Due to iatrogenic trauma (e.g. when operating for HOCM or resection of sub-valvular stenosis, or operating on the mitral valve).<br>20. **Failed TAVR**<br>21. **Failed TMVR**<br>22. **Functional tricuspid valve:** Normal leaflet anatomy with tricuspid regurgitation due to annular dilatation.<br>23. **Carcinoid syndrome:** Tricuspid regurgitation in patient with demonstrate carcinoid syndrome.<br>24. **Other**

Coronary artery bypass

**Intra-operative decision to graft coronary artery**	○Yes ○No			
**Internal thoracic artery (ITA) used**	○Yes ○No	*If Yes*	**LITA (left ITA)**	○Yes ○No
			If Yes **LITA Skeletonised**	○Yes ○No
			**RITA (right ITA)**	○Yes ○No
			If Yes **RITA Skeletonised**	○Yes ○No
**Number of radial artery conduits harvested**	○0 ○1 ○2			
**Number of distal arterial grafts (distal anastomoses with arterial conduits)**	○0 ○1 ○2 ○3 ○4 ○5 ○6 ○7 ○8 ○9			
**Number of ITA distal anastomoses**	○0 ○1 ○2 ○3 ○4 ○5 ○6			
**Number of radial distal anastomoses**	○0 ○1 ○2 ○3 ○4 ○5 ○6			
**Number of vein distal anastomoses**	○0 ○1 ○2 ○3 ○4 ○5 ○6 ○7 ○8 ○9			
**Number of GEPA distal anastomoses**	○0 ○1 ○2 ○3 ○4 ○5 ○6			
**Arterial T-graft or Y-grafts used**	○Yes ○No			

Valve surgery details

Aortic valve

**Stenosis**	○Yes ○No		
**Regurgitation / insufficiency**	○None ○Trivial ○Mild ○Moderate ○Severe		
**Pathology / aetiology**	○Rheumatic<br>○Myxomatous degeneration<br>○Peri-prosthetic leak<br>○Previous infection<br>○Other degenerative disease<br>○Trauma<br>○Other	○Congenital<br>○Failed prior repair<br>○Prosthetic valve thrombosis<br>○Marfans<br>○Dissection<br>○Iatrogenic	○Idiopathic Calcific<br>○Prosthetic valve failure<br>○Active infection<br>○Annuloaortic ectasia<br>○Tumour<br>○Failed TAVR

Mitral valve

**Stenosis**	○Yes ○No		
**Regurgitation / insufficiency**	○None ○Trivial ○Mild ○Moderate ○Severe		
**Pathology / aetiology**	○Rheumatic<br>○Idiopathic Calcific<br>○Prosthetic valve failure<br>○Active infection<br>○Other degenerative disease<br>○Iatrogenic	○Congenital<br>○Myxomatous degen.<br>○Peri-prosthetic leak<br>○Previous infection<br>○Tumour<br>○Failed TMVR	○Ischaemic<br>○Failed prior repair<br>○Prosthetic valve thrombosis<br>○Marfans<br>○Trauma<br>○Other

Tricuspid valve

**Stenosis**	○Yes ○No		
**Regurgitation / insufficiency**	○None ○Trivial ○Mild ○Moderate ○Severe		
**Pathology / aetiology**	○Rheumatic<br>○Failed prior repair<br>○Prosthetic valve thrombosis<br>○Marfans<br>○Trauma<br>○Carcinoid syndrome	○Congenital<br>○Prosthetic valve failure<br>○Active infection<br>○Other degenerative disease<br>○Iatrogenic<br>○Other	○Myxomatous degen.<br>○Peri-prosthetic leak<br>○Previous infection<br>○Tumour<br>○Functional

Pulmonary valve

**Stenosis**	○Yes ○No		
**Regurgitation / insufficiency**	○None ○Trivial ○Mild ○Moderate ○Severe		
**Pathology / aetiology**	○Rheumatic<br>○Failed prior repair<br>○Prosthetic valve thrombosis<br>○Other degenerative disease<br>○Iatrogenic	○Congenital<br>○Prosthetic valve failure<br>○Active infection<br>○Tumour<br>○Other	○Myxomatous degen.<br>○Peri-prosthetic leak<br>○Previous infection<br>○Trauma

Implant model	Enter the manufacturer's model number from the ANZSCTS database prosthesis list.
Serial	Enter the serial number of implanted prosthesis.
Lot number	Enter the lot number of implanted prosthesis.
Size	Enter the size of the prosthesis implant.
Explant model	Enter the manufacturer's model number for the explanted valve using the ANZSCTS database prosthesis list.
Serial	Enter the serial number of explanted prosthesis.
Size	Enter the size of the prosthesis explant.

**Post operative data**	
RBC	Were **allogeneic** red blood cells (RBC) transfused during the intra-operative or post-operative period? ***Do NOT include:*** *Pre-donated blood, Cell saver blood, Pump residual blood, Chest tube recirculated blood*
Bank RBC (units)	Enter the total number of RBC units transfused.
Non RBC	During the index admission, were blood products other than RBC (e.g. FFP and Platelets) transfused? ***Note: Excludes Albumin***
Platelets (units)	Indicate the number of platelet units transfused. ***Note: Indicate units and not pooled bags***
Novo 7 (units)	Indicate milligrams of Novo 7 used. ***Note: Novo7 comes in 1, 2, 4, and 8 mg vials. Dose administered is between 50-90mcg per kg. E.g. 50mcgx70kg person = 3500mcg=3.5mg.***
Fresh frozen plasma (unit)	Indicate the number of Fresh Frozen Plasma (FFP) units used.
Cryo (units)	Indicate the number of Cryoprecipitate units used.
ICU admission – date/time	Record the date and time of admission to ICU from OR after surgery.<br>***Note: This does not include ICU admission prior to surgery i.e. indicate the date/time patient was admitted to ICU after surgery (regardless of other time/date of previous ICU admission).***<br>Enter **09/09/9999 00:00** for unknown date/time
Extubation - date/time	Record the date and time of extubation after surgery.<br>***Note: Ventilation time should be when the patient is attached to a ventilator including the weaning process but does not include spontaneous ventilation through a tracheostomy.***<br>Enter **09/09/9999 00:00** for unknown date/time.
ICU discharge - date/time	Record the date and time of discharge from ICU.<br>***Note: This includes discharge to high dependency unit (HDU), general ward or death.***<br>Enter **09/09/999 00:00** for unknown date/time.
Readmitted to ICU	During the index operation, was the patient readmitted to ICU following the initial discharge from ICU to HDU or general ward?<br>***Note: This does not include patients initially in ICU and returned to theatre for complications.***
Reintubation	During the index admission, was the patient was re-intubated after the initial extubation?<br>***Note: This does not include patients who were intubated for a subsequent surgical procedure.***
Reintubation - date/time	Record the date and time when the patient was re-intubated. Enter **09/09/9999 00:00** for unknown date/time.
Re-extubation - date/time	Record the date and time when the patient was extubated following re-intubation.<br>Enter **09/09/9999 00:00** for unknown date/time.
ICC loss (first 4 hours post surgery)	Record the fluid loss (in millilitres) from the pericardial/mediastinal (intercostal catheter) drains in the first 4 hours post-operation.

Valve surgery (continued from page 8)

Aortic valve procedure
- ○ No
- ○ Replacement
- ○ Root Reconstruction with Valve Conduit (Bentall)
- ○ Root Reconstruction with Valve Sparing (David)
- ○ Resuspension of the Aortic Valve
- ○ Resection of Sub-Aortic Stenosis
- ○ Repair paravalvular leak
- ○ Ross procedure
- ○ Inspection only
- ○ Decalcification of valve only
- ○ Subcommissural annuloplasty
- ○ Thrombus removal
- ○ Root enlargement (Manougian type excludes Nicks)
- ○ TAVR
- ○ Aortic valvuloplasty with subcommissural annuloplasty
- ○ Aortic valvuloplasty without subcommissural annuloplasty
- ○ Valvotomy with annuloplasty ring
- ○ Valvotomy without annuloplasty ring
- ○ Tumor tissue removal
- ○ Other

Implant model ______
Serial no ______
Size ____ mm **Lot number** ______
Explant model ______
Serial no ______
Size ____ mm

Mitral valve procedure
- ○ No
- ○ Annuloplasty only
- ○ Replacement
- ○ Repair/Reconstruction with annuloplasty
- ○ Repair/Reconstruction without annuloplasty
- ○ Commissurotomy with annuloplasty ring
- ○ Commissurotomy without annuloplasty ring
- ○ Repair paravalvular leak
- ○ Inspection only
- ○ Decalcification of valve only
- ○ Thrombus removal
- ○ Alfieri Suture
- ○ Tumor tissue removal
- ○ Insertion of Mitraclip device
- ○ TMVR

Implant model ______
Serial no ______
Size ____ mm **Lot number** ______
Explant model ______
Serial no ______
Size ____ mm

Tricuspid valve procedure
- ○ No
- ○ Annuloplasty only
- ○ Replacement
- ○ Repair/Reconstruction with annuloplasty
- ○ Repair/Reconstruction without annuloplasty
- ○ Commissurotomy with annuloplasty ring
- ○ Commissurotomy without annuloplasty ring
- ○ Repair paravalvular leak
- ○ Valvectomy (no replacement)
- ○ Inspection only
- ○ Thrombus removal
- ○ Tumor tissue removal

Implant model ______
Serial no ______
Size ____ mm **Lot number** ______
Explant model ______
Serial no ______
Size ____ mm

Pulmonary valve procedure
- ○ No
- ○ Replacement
- ○ Repair paravalvular leak
- ○ Inspection only
- ○ Replacement of pulmonary root as part of Ross procedure
- ○ Valvotomy with annuloplasty ring
- ○ Valvotomy without annuloplasty ring
- ○ Tumour tissue removal

Implant model ______
Serial no ______
Size ____ mm **Lot number** ______
Explant model ______
Serial no ______
Size ____ mm

**Complications**	
Return to theatre	Did the patient return to the operating theatre? ***Note: This includes operative procedures done in the ICU that would normally be performed in the operating room.***
Valve dysfunction	Did the patient return to theatre for valve dysfunction?
Bleeding/tamponade	Did the patient return to theatre for bleeding/tamponade?
Graft occlusion	Did the patient return to theatre for:<br>a. Graft refashion **OR**<br>b. Grafting of a previously ungrafted coronary.
Deep sternal infection	Did the patient return to theatre for infection of sternal bone muscle and/or mediastinum?
Deept thoracotomy infection	Did the patient return to theatre for infection involving a thoracotomy or parasternal site?
Insertion of pacemaker	Did the patient return to theatre for insertion of pacemaker or AICD?
Other cardiac	Did the patient return to theatre for other cardiac reasons (excluding insertion of pacemaker or AICD)?
Other non cardiac	Did the patient return to theatre for other non-cardiac reasons?
New renal failure	Was there acute post-operative renal insufficiency characterised by one of the following:<br>a. Increased serum creatinine to >0.2mmol/l (>200mol/l) AND a doubling or greater increasein creatinine over the baseline pre-operative value AND the patient did not require pre- operative dialysis/haemofiltration<br>OR<br>b. A new post-operative requirement for dialysis/haemofiltration (when the patient did not require this pre-operatively).<br>***Note: Renal insufficiency must not be present pre-operatively. Pre-operative renal transplant does not count as renal insufficiency if the patient did not have impaired kidney function and did not require dialysis/haemofiltration.***
Haemofiltration	Did the patient undergo acute institution of haemofiltration (or dialysis) as treatment for **new** renal failure?<br>***Note: this excludes haemofiltration for removal of fluid with normal serum urea and creatinine***
Highest post-op creatinine level	Record the highest serum creatinine level recorded after surgery.<br>20 mol/L to 2000 mol/L
Acute myocardial infarction	Was a peri-/post- operative myocardial infarction (MI) diagnosed by finding at **least two** of the following criteria:<br>a. Enzyme level elevation either:<br>▪ CK-MB >30 units **OR**<br>▪ Troponin > 20.0 micrograms/L **OR**<br>▪ Troponin level equivalent documented at your instruction, provided operation does not involve myocardial incision.<br>b. New wall motion abnormalities<br>c. Serial ECG (at least two) showing Q waves, duration ≥0.03ms in 2 contiguousleads.
Cardiogenic shock	Did the patient suffer peri-/ post- operative cardiogenic shock?<br>**Note:** Only code yes if all of the following criteria apply:<br>1. Sustained (>30 minutes) episode of systolic blood pressure <90mm Hg or the requirement for parenteral inotropic or vasopressor agents or mechanical support (e.g. Intra-aortic balloon pump (IABP), extracorporeal circulation, ventricular assist devices to maintain BP > 90mm Hg); **AND**<br>2. Evidence of elevated filling pressures (e.g. pulmonary congestion on examination or chest radiograph); **AND**<br>3. Evidence of end organ hypoperfusion (e.g. urine output 30mL/hours, or cold/diaphoretic extremities, or obtunded mental status if previously normal, etc.).
Post-operative haemoglobin	Record the **lowest** haemoglobin recorded post-operatively.<br>**Range:** 40g/L to 200g/L
Cardiac inotrope use – for longer than 4 hours post-operatively	Was an inotrope used to maintain cardiac output or SVR for longer than 4 hours post-operatively?<br>***Note: Include Dopamine at > 300 µg/min. Do NOT include routinely administered Milrinone***
Cardiac inotrope use - for low cardiac output syndrome	Were inotrope(s) used for low cardiac output syndrome longer than four hours post-operatively (i.e. when an inotrope is administered with the intent to improve cardiac output irrespective of the reason for that decision)?<br>***Note: Do NOT include routinely administered Milrinone.***
Cardiac vasopressor use - for low SVR syndrome	Was a vasopressor used for low systemic vascular resistance syndrome for longer than 4 hours post-operatively (i.e. when a primarily alpha-adrenergic agonist is given with the intent to increase SVR (where SVR < 800))?<br>***Note: This is usually in the presence of high cardiac output, however, does not include Noradrenaline given with Milrinone.***

Post operative data

**RBC**	○Yes ○No	*If Yes*	**Bank RBC**	☐☐	units
**Non RBC**	○Yes ○No	*If Yes*	**Platelets**	☐☐	units
			**Novo 7**	☐☐	mg
			**Fresh frozen plasma**	☐☐	units
			**Cryo**	☐☐	units
**ICU admission**	☐☐ / ☐☐ / ☐☐☐☐ ☐☐ : ☐☐ ○AM ○PM		*OR*	◻No ICU admission	
**Extubation**	☐☐ / ☐☐ / ☐☐☐☐ ☐☐ : ☐☐ ○AM ○PM				
**ICU discharge**	☐☐ / ☐☐ / ☐☐☐☐ ☐☐ : ☐☐ ○AM ○PM				
**Readmitted to ICU**	○Yes ○No				
**Reintubation**	○Yes ○No	*If Yes*	**Reintubation Date/time**	☐☐ / ☐☐ / ☐☐☐☐ ☐☐ : ☐☐ ○AM ○PM	
			**Re-extubation Date/time**	☐☐ / ☐☐ / ☐☐☐☐ ☐☐ : ☐☐ ○AM ○PM	
**ICC loss - first 4 hours post surgery**	☐☐☐☐ mls				

Complications

**Return to theatre**	○Yes ○No	*If Yes*	**Valve dysfunction**	○Yes ○No
			**Bleeding/tamponade**	○Yes ○No
			**Graft occlusion**	○Yes ○No
			**Deep sternal infection**	○Yes ○No
			**Deep thorocotomy wound infection**	○Yes ○No
			**Other cardiac**	○Yes ○No
			**Other non cardiac**	○Yes ○No
			**Insertion of pacemaker**	○Yes ○No

Renal

**New renal failure**	○Yes ○No	*If Yes*	**Haemofiltration**	○Yes ○No
**Highest post-op creatinine level**	☐☐☐☐	(20 to 2000 μmol/L)	OR	◻Unknown

Peri/Post Operative

**Acute myocardial infarction**	○Yes ○No	
**Cardiogenic shock**	○Yes ○No	
**Post-operative haemoglobin**	☐☐☐	(40 to 200 g/L)

Cardiac

**Cardiac inotrope use – for longer than 4 hours post-operatively**	○Yes ○No
**Cardiac inotrope use - for low cardiac output syndrome**	○Yes ○No
**Cardiac vasopressor use - for low SVR syndrome**	○Yes ○No

**Complications ctd.**	
New cardiac arrhythmia	Did any new form of cardiac arrhythmia occur that required treatment?<br>***Note: Includes brady and tachy arrhythmias of atrial or ventricular origin and any A-V conduction disturbances or requirement for permanent pacemaker insertion.***
New heart block (requiring PPM)	Did the patient develop new heart block requiring the implantation of a permanent pacemaker prior to discharge?
New other brady-arrhythmia (requiring PPM)	Did the patient develop a new bradyarrhythmia not otherwise specified requiring the implantation of permanent pacemaker prior to discharge?
Cardiac arrest	Did the patient have a new cardiac arrest documented by one of the following:<br>• Ventricular fibrillation, Rapid ventricular tachycardia with haemodynamic instability, asystole, Pulseless electrical activity (PEA)?
Atrial fibrillation or flutter (requiring Rx)	Was there a new onset of atrial fibrillation/flutter (AF) requiring treatment? ***Note: Does not include recurrence of pre-op AF.***
New ventricular tachycardia	Did any new form of ventricular tachycardia (greater than 6 beat run) occur that required treatment?
Stroke permanent	Did the patient experience a stroke or new central neurologic deficit (persisting for >72 hours) peri- or post-operatively? ***Note: Neurological deficit is characterised by persistent loss of neurological function caused by an ischaemic or haemorrhagic event.***
Stroke transient	Did the patient experience a new transient central neurologic deficit that was resolved completely within 72 hours (TIA/RIND)?
New continuous coma >=24 hours	Did the patient have post-operative coma of neurologic aetiology lasting at least 24 hours?<br>***Note: This excludes metabolic and drug related coma***.
Prolonged ventilation > 24 hours	Was the patient on prolonged ventilation post-operatively for pulmonary insufficiency requiring ventilator support –includes (but not limited to) causes such as Adult Respiratory Distress Syndrome (ARDS) and pulmonary oedema –for a total period of longer than 24 hours? Use **cumulative period** if patient is re-intubated.
Pulmonary embolism	Did the patient have a new pulmonary embolism diagnosed by study such as ventilation/perfusion (V/Q) scan or angiogram?
Pneumonia	Was pneumonia diagnosed post-operatively by one of the following:<br>a. Positive cultures of sputum or trans-tracheal aspirate **OR**<br>b. Clinical, including haematological findings consistent with the diagnosis of pneumonia and radiographic evidence
Pneumothorax	Indicate whether the patient had a post-operative pneumothorax requiring intervention. **Note:** Interventions include chest tube insertion, needle aspiration or other invasive procedure. Do not capture a small pneumothorax followed with serial chest X-rays.
Pleural effusion	Indicate whether a post-operative pleural effusion required drainage via thoracentesis or chest tube insertion. **Note:** Interventions include chest tube insertion, needle aspiration or other invasive procedure. May include hemothorax.
Deep sternal wound infection	Did the patient develop infection of sternal bone, muscle and/or mediastinum? The patient must have **wound debridement and** one of the following:<br>a. Positive cultures<br>b. Treatment with antibiotics
Superficial access wound infection	Did the patient develop an infection involving the skin and subcutaneous tissues of the incision occurring <u>within 30-days</u> post-operation?<br>***For this diagnosis to be made, ONE of the following must be present:***<br>a. Purulent drainage from the superficial incision.<br>b. Organisms isolated from an aseptically obtained culture of fluid or tissue from the superficial incision.<br>c. Superficial incision deliberately opened by surgeon **whether or not** it is culture positive.<br>d. Diagnosis of superficial incisional surgical site infection by operating surgeon or other attending clinician
Donor site deep wound infection	Did the patient develop an infection involving deep soft tissues (e.g. fascial and muscle layers and/or organs/spaces opened or manipulated during surgery) occurring within <u>30 days</u> after the operative procedure if implant not present?<br>***For this diagnosis to be made, ONE of the following must be present:***<br>a. Purulent drainage from deep soft tissue but not from the organ/space component of the surgical site.<br>b. Spontaneous dehiscence at incision site or the wound is deliberately explored by a surgeon with the patient showing evidence of one or more of the following signs or symptoms.<br>• Fever > 38˚C, localised pain or tenderness with culture-positive specimen. A culture- negative finding does not meet this criterion unless the patient was on antibiotics immediately prior to the wound being explored and/or the culture being taken.<br>• Organisms isolated from an aseptically obtained culture of fluid or tissue obtained from an organ/space.<br>• An abscess or other evidence of infection involving a deep/organ space found on direct examination, during reoperation, or by histopathologic or radiologic examination.<br>c. Diagnosis of or antimicrobial treatment of a deep incisional or organ/space surgical site infection by operating surgeon or assisting physician.
Deep thoracotomy wound infection	Did the patient develop an infection involving a thoracotomy or parasternal site?<br>***For this diagnosis to be made, ONE of the following must be present:***<br>a. Wound opened with excision of tissue<br>b. Positive culture<br>c. Treatment with antibiotics
Septicaemia	Did the patient develop septicaemia defined by positive blood cultures supported by at least two of the following indices of clinical infection:<br>a. Fever<br>b. Elevated granulocyte cell counts<br>c. Elevated and increasing C-reactive protein (CRP)<br>d. Elevated and increasing erythrocyte sedimentation rate (ESR) post-operatively.
Leg or arm	'**Donor site superficial wound infection'**<br>Did the patient develop an infection involving the skin and subcutaneous tissues of the donor site/s occurring <u>within 30-days</u> post-operation?<br>***For this diagnosis to be made, ONE of the following must be present:***<br>a. Purulent drainage from the superficial incision.<br>b. Organisms isolated from an aseptically obtained culture of fluid or tissue from the superficial incision.<br>c. Superficial incision deliberately opened by surgeon **whether or not** it is culture positive.<br>d. Diagnosis of superficial incisional surgical site infection by operating surgeon or other attending clinician
Aortic dissection	Did dissection occur in any part of the aorta?
Acute limb ischaemia	Was there any evidence of limb ischaemia?
Anticoagulant complications	Did the patient develop any bleeding, haemorrhage, and/or embolic events related to anticoagulant therapy?

Complications (continued from page 10)

Arrhythmia

**New cardiac arrhythmia**	○Yes	○No	*If Yes*	**New heart block (requiring PPM)**	○Yes	○No
				**New other brady-arrhythmia (requiring PPM)**	○Yes	○No
				**Cardiac arrest**	○Yes	○No
				**Atrial fibrillation or flutter (requiring Rx)**	○Yes	○No
				**New ventricular tachycardia**	○Yes	○No

Neurological

**Stroke permanent**	○Yes	○No
**Stroke transient**	○Yes	○No
**New continuous coma >=24 hours**	○Yes	○No

Pulmonary

**Prolonged ventilation > 24 hours**	○Yes	○No
**Pulmonary embolism**	○Yes	○No
**Pneumonia**	○Yes	○No
**Pneumothorax**	○Yes	○No
**Pleural effusion**	○Yes	○No

COVID-19

**Post operative diagnosis of COVID-19**	○Yes	○No

Infection

**Deep sternal wound infection**	○Yes	○No
**Superficial access wound infection**	○Yes	○No
**Donor site deep wound infection**	○Yes	○No
**Deep thoracotomy wound infection**	○Yes	○No
**Septicaemia**	○Yes	○No
**Leg or arm**	○Yes	○No

Vascular

**Aortic dissection**	○Yes	○No	
**Acute limb ischaemia**	○None	○Upper	○Lower

Other

**Anticoagulant complications**	○Yes	○No
**GIT complications**	○Yes	○No
**Multi -system failure**	○Yes	○No

Complications ctd.	
GIT complications	Did the patient develop any GIT complication post-operatively including any of the following:

	1. GI bleeding requiring transfusion<br>2. Pancreatitis with abnormal amylase/lipase requiring nasogastric suction therapy<br>3. Cholecystitis requiring cholecystectomy or drainage<br>4. Mesenteric ischaemia requiring exploration<br>5. Hepatitis<br>6. GI complication<br>7. Other
Multi -system failure	Did the patient develop multi-system failure post-operatively?<br>***For this diagnosis to be made, TWO or more of the following major organ systems must fail concurrently for at least 48 hours:***<br>a. Renal –new renal failure (previously defined)<br>b. Respiratory –requires endotracheal intubation for respiratory dysfunction<br>c. Cardiac –the use of inotropes and/or IABP to treat low cardiac output<br>d. Hepatic failure on the basis of enzymes, and bilirubin estimation.

**Discharge**	
Cognisant patient withdraws from treatment	Did the patient who was aware of the consequences of his/her actions, elect to withdraw treatment in circumstances where they would survive if treatment had continued.<br>***Note: Completing 'YES' to this field implies automatic review of patient's hospital file and permission for ANZSCTS personnel to review their case.***
Discharge location	Indicate from the list the location where the patient was discharged to (from the hospital following the admission during where the index operation was performed).<br>1. **Home:** discharged home with no planned contact before routine review<br>2. **Hospital in the home:** discharged home with planned visits to home by medical or paramedical staff<br>3. **Rehabhilitation unit / Hosptial:** Discharged for inpatient rehabilitation.<br>4. **Local or referring hospital:** discharged to hospital for continuing acute care.<br>5. Hospital mortality<br>6. **Other cardiac unit**: i.e. transferred to another hospital for further cardiac surgical intervention (eg. ECMO or cardiac transplant).
**Mortality**	
Mortality post discharge	Did the patient die after discharge from hospital but within 30 days of surgery?
Mortality - date	Indicate the date of death if the patient:<br>a. Died within 30 days from date of surgery (whether discharged or not)<br>b. Died in hospital (during the index admission at any time).
Mortality - location	Specify the location of patient mortality:<br>1. Operating room<br>2. Hospital in which the operation was performed (other than operating room. Eg. ICU or ward)<br>3. Home (including hospital in the home)<br>4. Other care facility
Mortality - primary cause	Indicate the PRIMARY cause of death, i.e. the first significant event which ultimately led to death.<br>Select from **ONE** of the following:<br>1. Cardiac cause<br>2. Neurologic event<br>3. Renal failure<br>4. Vascular event (peripheral vascular or aortic but not aortic dissection)<br>5. Infection<br>6. Respiratory failure<br>7. Valvular dysfunction<br>8. Multisystem failure (as defined in above)<br>9. Pulmonary embolism<br>10. Aortic dissection<br>11. Other<br>12. Unknown
Mortality - subsequent cause	Specific cause of death.<br>This applies only when **PRIMARY** cause of death is cardiac or infection.<br>***If PRIMARY cause is cardiac, select from:***<br>1. Ischaemic/<br>2. Other Cardiac<br>3. Unknown<br>***If PRIMARY cause if infection, selection from:***<br>1. Septicaemia<br>2. Endocarditis<br>3. Other infection<br>4. Unknown

Discharge		
**Cognisant patient withdraws from treatment**	○Yes ○No	
**Discharge location**	○Home<br>○Rehabilitation Unit/Hospital<br>○Hospital mortality	○Hospital in the Home (HITH)<br>○Local or referring hospital<br>○Other cardiac unit for further CS intervention

Mortality				
**Mortality post-discharge**	○Yes ○No ○Unknown			
**Mortality - date**	☐☐ / ☐☐ / ☐☐☐☐			
**Mortality - location**	○Operating Room ○Hospital ○Home/HITH ○Other Facility			
**Mortality - primary cause**	○Cardiac<br>○Neurologic<br>○Renal<br>○Vascular<br>○Infection<br>○Respiratory failure<br>○Valvular<br>○Multisystem failure<br>○Pulmonary embolism<br>○Aortic dissection<br>○Other<br>○Unknown	*If Cardiac*	**Specify**	○Ischemia<br>○Other Cardiac<br>○Unknown
		If Infection	**Specify**	○Septicaemia<br>○Endocarditis<br>○Other Infection<br>○Unknown

Readmission					
**Readmission ≤30 days from surgery**	○Yes	○No	*If Yes*	**Anticoagulant complication**	○Yes ○No
				**Pneumonia or other respiratory complication**	○Yes ○No
				**Myocardial infarction (MI)**	○Yes ○No ○Unknown
				**Recurrent angina**	○Yes ○No
				**Readmission unrelated to cardiac surgery**	○Yes ○No
				**Arrhythmia**	○Yes ○No
				**Congestive heart failure (CHF)**	○Yes ○No
				**Valve dysfunction**	○Yes ○No
				**Pericardial effusion**	○Yes ○No
				**Cardiac tamponade**	○Yes ○No ○Unknown
				**Pleural effusion**	○Yes ○No
				**Other complication related to cardiac surgery**	○Yes ○No
				**Deep sternal infection**	○Yes ○No
				**Incisional complication**	○Yes ○No
**Readmission 31 - 90 days from surgery**	○Yes	○No	*If Yes*	**Anticoagulant complication**	○Yes ○No
				**Pneumonia or other respiratory complication**	○Yes ○No
				**Myocardial infarction (MI)**	○Yes ○No ○Unknown
				**Recurrent angina**	○Yes ○No
				**Readmission unrelated to cardiac surgery**	○Yes ○No
				**Arrhythmia**	○Yes ○No
				**Congestive heart failure (CHF)**	○Yes ○No
				**Valve dysfunction**	○Yes ○No
				**Pericardial effusion**	○Yes ○No
				**Cardiac tamponade**	○Yes ○No ○Unknown
				**Pleural effusion**	○Yes ○No
				**Other complication related to cardiac surgery**	○Yes ○No
				**Deep sternal infection**	○Yes ○No
				**Incisional complication**	○Yes ○No

**Readmission**	
Readmission <=30 days from surgery	Was the patient readmitted as an inpatient within 30 days from the date of surgery for ANY reason?<br>***Note: Readmission means admission to general hospital not emergency, short-stay wards or planned transfer to rehabilitation facility. Date of surgery counts as day zero.***
Anticoagulant complication	Was the patient readmitted as an inpatient within 30 days from surgery due to an anticoagulant complication (i.e. haemorrhage associated with **demonstrated** over-anticoagulation or thrombosis or embolism associated with **demonstrated** under anticoagulation)?
Pneumonia or other respiratory complication	Was the patient readmitted as an inpatient within 30 days from surgery for pneumonia or other respiratory complication?<br>Pneumonia is diagnosed by the following:<br>a. Positive cultures of sputum or trans-tracheal aspirate **OR**<br>b. Clinical, including haematological findings consistent with the diagnosis of pneumonia and radiographic evidence
Myocardial infarction (MI)	Was the patient readmitted as an inpatient within 30 days from surgery for myocardial infarction (MI), diagnosed according to the definition listed on above?
Recurrent angina	Was the patient readmitted as an inpatient within 30 days from the date of surgery for recurrent angina? Objective confirmation that chest pain is due to ischaemia by exercise test (ECG, nuclear, echo, exercise test or angiography) is required to meet this diagnosis.
Readmission unrelated to cardiac surgery	Was the patient readmitted as an inpatient within 30 days from surgery due to reasons not related to cardiac surgery?
Arrhythmia	Was the patient readmitted as an inpatient within 30 days from surgery for management of any arrhythmia?
Congestive heart failure (CHF)	Was the patient readmitted as an inpatient within 30 days from surgery for treatment of congestive heart failure (CHF) as evidenced by one (or more) of the following:<br>a. Paroxysmal nocturnal dyspnoea (PND)<br>b. Deteriorating dyspnoea on exertion (DOE) due to heart failure<br>Chest x-ray (CXR) showing pulmonary congestion.
Valve dysfunction	Was the patient readmitted as an inpatient within 30 days from surgery for valve dysfunction?
Pericardial effusion	Was the patient readmitted as an inpatient within 30 days from surgery for treatment of pericardial effusion?<br>***Note: If the effusion (of whatever nature, i.e. serous, bloody, infective) has NOT required draining and/or caused tamponade then indicate other complications relating to cardiac surgery.***
Cardiac tamponade	Was the patient readmitted as an inpatient within 30 days from surgery for cardiac tamponade?<br>***Note: If the pericardial effusion has caused cardiac tamponade which is a <u>physiological</u> diagnosis, then indicate cardiac tamponade only***
Pleural effusion	Was the patient readmitted as an inpatient within 30 days from surgery for symptomatic pleural effusion?
Other complication related to cardiac surgery	Was the patient readmitted as an inpatient within 30 days from surgery for treatment for an 'other' complication (not specified in 130.1-130.7) related to cardiac surgery (e.g. renal, hepatic, GI, etc.)?
Deep sternal infection	Was the patient readmitted as an inpatient within 30 days from surgery for deep sternal wound infection?<br>***Note: Deep sternal wound infection involves muscle and bone, with or without mediastinal involvement and demonstrated by surgical exploration. Must have wound debridement and one of the following:***<br>a. ***Positive culture***<br>b. ***Treatment with antibiotics***
Incisional complication	Was the patient readmitted as an inpatient within 30 days from surgery for a complication involving the saphenous vein, radial artery harvest, thoracotomy or **non-sternotomy** cardiac access incision?
**Readmission 31 -90 days**	
Readmission in 31 - 90 days from date of surgery	Was the patient readmitted as an inpatient between 31 and 90 days from the date of surgery for ANY reason?<br>***Note: Readmission means admission to general hospital not emergency, short-stay wards or planned transfer to rehabilitation facility. Date of surgery counts as day zero.***
Anticoagulant complication	Was the patient readmitted as an inpatient between 31 and 90 days from surgery due to an anticoagulant complication (i.e. haemorrhage associated with **demonstrated** over-anticoagulation or thrombosis or embolism associated with **demonstrated** under anticoagulation)?
Pneumonia or other respiratory complication	Was the patient readmitted as an inpatient between 31 and 90 days from surgery for pneumonia or other respiratory complication?<br>Pneumonia is diagnosed by the following:<br>a. Positive cultures of sputum or trans-tracheal aspirate **OR**<br>b. Clinical, including haematological findings consistent with the diagnosis of pneumonia and radiographic evidence
Myocardial infarction (MI)	Was the patient readmitted as an inpatient between 31 and 90 days from surgery for myocardial infarction (MI), diagnosed according to the definition listed on above?
Recurrent angina	Was the patient readmitted as an inpatient between 31 and 90 days from the date of surgery for recurrent angina? Objective confirmation that chest pain is due to ischaemia by exercise test (ECG, nuclear, echo, exercise test or angiography) is required to meet this diagnosis.
Readmission unrelated to cardiac surgery	Was the patient readmitted as an inpatient between 31 and 90 days from surgery due to reasons not related to cardiac surgery?
Arrhythmia	Was the patient readmitted as an inpatient between 31 and 90 days from surgery for management of any arrhythmia?
Congestive heart failure (CHF)	Was the patient readmitted as an inpatient between 31 and 90 days from surgery for treatment of congestive heart failure (CHF) as evidenced by one (or more) of the following:<br>a. Paroxysmal nocturnal dyspnoea (PND)<br>b. Deteriorating dyspnoea on exertion (DOE) due to heart failure<br>c. Chest x-ray (CXR) showing pulmonary congestion.
Valve dysfunction	Was the patient readmitted as an inpatient between 31 and 90 days from surgery for valve dysfunction?
Pericardial effusion	Was the patient readmitted as an inpatient between 31 and 90 days from surgery for treatment of pericardial effusion?<br>***Note: If the effusion (of whatever nature, i.e. serous, bloody, infective) has NOT required draining and/or caused tamponade then indicate other complications relating to cardiac surgery.***
Cardiac tamponade	Was the patient readmitted as an inpatient between 31 and 90 days from surgery for cardiac tamponade?<br>***Note: If the pericardial effusion has caused cardiac tamponade which is a <u>physiological</u> diagnosis, then indicate cardiac tamponade only***
Pleural effusion	Was the patient readmitted as an inpatient between 31 and 90 days from surgery for symptomatic pleural effusion?
Deep sternal infection	Was the patient readmitted as an inpatient between 31 and 90 days from surgery for deep sternal wound infection?<br>***Note: Deep sternal wound infection involves muscle and bone, with or without mediastinal involvement and demonstrated by surgical exploration. Must have wound debridement and one of the following:***<br>***a. Positive culture***<br>***b. Treatment with antibiotics***
Incisional complication	Was the patient readmitted as an inpatient between 31 and 90 days from surgery for a complication involving the saphenous vein, radial artery harvest, thoracotomy or **non-sternotomy** cardiac access incision?
Other complication related to cardiac surgery	Was the patient readmitted as an inpatient between 31 and 90 days from surgery for treatment for an 'other' complication (not specified) related to cardiac surgery (e.g. renal, hepatic, GI, etc.)?

Anaesthetic audit

Staff					
**Consultant**					
**Registrar**					
**Perfusionist**					
Monitors					
**PAC**	○Yes ○No				
**TOE**	○Yes ○No				
TOE					
**Performed by**					
**Complications**					
**Change of management**	○Yes ○No	*If Yes*	**Change valve**	○Yes	○No
			**Assist de-airing**	○Yes	○No
			**Added inotrope**	○Yes	○No
			**Fluid management**	○Yes	○No
			**New pathology**	○Yes	○No
Lines					
**CVL - Carotid puncture**	○Yes ○No	*If Yes*	**Needle size**		
**CVL - Carotid dilated**	○Yes ○No				
Antifibrinolytics					
**Tranexamic acid**	○Yes ○No				
**Epsilon aminocaproic acid (EACA)**	○Yes ○No				
Incidents					
**Anaesthetic/perfusion incidents occurred?**	○Yes ○No	*If Yes* **Specify**			

----END OF FORM----

CARDIAC SURGERY DATA COLLECTION FORM
