# Supplementary material for: Formative research for a pre-operative psychosocial screening program for cardiac surgical patients: The EMBRACE study, a mixed methods knowledge to action protocol
Source: PLoS One. 2025 Dec 30;20(12):e0322592. doi: 10.1371/journal.pone.0322592 (PMC12752988; doi:10.1371/journal.pone.0322592)
Supplement: S1 Letter — (PDF) [file pone.0322592.s003.pdf]

The Prince Charles Hospital
Metro North Hospital & Health Service

Queensland Government

Letter of invitation to participate in a research project

Dear Patient

The Prince Charles Hospital Department of Cardiothoracic Surgery in collaboration with the Department of Anaesthesia and Queensland University of Technology is conducting a research project called 'EMBRACE -Well-being support in cardiac surgery' to study how the sense of emotional well-being and stress might affect how patients recover from their operation and the chance of complications during or after their surgery. The study also aims to test if screening can be easily performed on a small electronic device to identify patients who might benefit, and what preferences they might have for well-being support during their cardiac surgical admission. This letter is an invitation to consider participating in the study.

We hope that the results of this study will improve our understanding of the relationship of cardiac surgical patients' emotional well-being and stress management with their health outcomes after surgery. The study will also help to determine patients' preferences for screening and well-being support during the surgical care period. If we understand who might be more at risk from surgery with respect to this, better support strategies can be put in place, and future patients can be offered additional help to improve their outcomes from their surgery. I have attached a detailed information sheet about the research project. Please read this information carefully and bring it with you when you visit the hospital for your surgery.

Consent

If you are willing to participate in the research project you will have the study explained to you and be asked to sign a consent form when you arrive at the hospital. A member of the research team will be available at the clinic if you have any concerns.

If you have any questions regarding the research project, please do not hesitate to phone 3139 4230 between the hours of 9.00 am to 5.00pm Monday to Wednesday and Friday.

Thank you from the research team.

Yours sincerely

Susan Smith, Clinical Research Coordinator and
the Cardiac Surgery and Anaesthetics Care Team
