# Supplementary material for: Formative research for a pre-operative psychosocial screening program for cardiac surgical patients: The EMBRACE study, a mixed methods knowledge to action protocol
Source: PLoS One. 2025 Dec 30;20(12):e0322592. doi: 10.1371/journal.pone.0322592 (PMC12752988; doi:10.1371/journal.pone.0322592)
Supplement: S1 PICF — (PDF) [file pone.0322592.s004.pdf]

Participant Information Sheet/Consent Form

Observational Study - *Adult providing own consent*

**Title**	Exploring the relationship between EMotional well-Being with health outcomes and patient pReferences for resources and support in cArdiaC surgEry. (EMBRACE)
**Short Title**	EMBRACE -Well-being support in cardiac surgery
**NEAF Protocol Number**	80524
**Principal Investigators**	Ms Susan Smith, Dr Esben Strodl, Dr Tricia Rolls,
**Associate Investigator(s)**	Dr Rishendran Naidoo, Dr Usha Gurunathan, Dr Marlien Varnfield, Professor David Kavanagh, Professor Jed Duff, Bo Janoschka, Dr Karen Hay.
**Location**	The Prince Charles Hospital

Part 1 What does my participation involve?

1 Introduction

You are invited to participate in this research project because you are going to have heart surgery. Heart surgery can be a stressful time and this research project is studying how the sense of emotional well-being and stress might affect how patients recover from that operation and if they are likely to have any complications during or after their surgery. The study also aims to test if screening can be easily performed on a small electronic device to identify people who might benefit from support and what preferences patients might have for their well-being support during the cardiac surgical admission.

This Participant Information Sheet/Consent Form tells you about the research project. It explains the tests and treatments involved. Knowing what is involved will help you decide if you want to take part in the research.

Please read this information carefully. Ask questions about anything that you don't understand or want to know more about. Before deciding whether to take part or not, you might want to talk about it with a relative, friend or your local doctor.

Participation in this research is voluntary. If you don't wish to take part, you don't have to. You will receive the best possible care whether you take part or not.

If you decide you want to take part in the research project, you will be asked to sign the consent section. By signing it you are telling us that you:

- Understand what you have read
- Consent to take part in the research project
- Consent to undertake surveys, questionnaires, and interviews as are described
- Consent to the use of your personal and health information as described.

You will be given a copy of this Participant Information and Consent Form to keep.

2 What is the purpose of this research?

There is already good evidence that the sense of well-being and stress management are related to both the development and progression of cardiac disease and also to health outcomes after surgery. The National Heart Foundation of Australia suggests that all cardiac patients should be screened to ensure best care can be provided. But the relationships between emotional well-being, stress management and cardiac surgical outcomes are complex and not well understood. Furthermore, an effective method for screening to identify people who might benefit from support has also not been determined. This means that screening questionnaire options must be tested to see if and how they could be used to effectively measure a patient's risk for surgical outcomes.

We hope that the results of this study will improve our understanding of the relationship of cardiac surgical patients' emotional well-being and levels of distress with their health outcomes after surgery. The study will also help to determine patients' preferences for screening and well-being support during the surgical care period. If we understand who might be more at risk from surgery with respect to this, better support strategies can be put in place, and future patients can be offered additional help to improve their outcomes from their surgery.

This research has been initiated by the study Principal Investigator Ms Susan Smith, Clinical Research Coordinator for the Cardiothoracic Surgery service at The Prince Charles Hospital as part of studies to obtain a PhD degree. It is being undertaken by a group of investigators which also includes Queensland University of Technology Psychologist, Associate Professor Esben Strodl, The Prince Charles Hospital Director of Psychology Dr Tricia Rolls, and the Cardiothoracic Surgery and Anaesthetics medical team at The Prince Charles Hospital.

This study has been funded by a New Investigator Grant provided by The Prince Charles Hospital Foundation and by a post-graduate student allocation from QUT.

3 & 4 What does participation in this research involve, what do I have to do?

To participate in this study, we would ask for your signed consent before any study assessments are performed. If you give consent you would be participating in a single centre, non-randomised, non-controlled and prospective research study. This means that everyone at Prince Charles Hospital who is eligible and gives study consent will have the same assessments performed before their surgery and health outcomes measured during recovery. We will invite all adult patients booked for non-emergency cardiac surgery at The Prince Charles Hospital to participate if their surgeon is also agreeable.

Participation in this research would involve some extra assessment taken during your hospital visit prior to your surgery or immediately after admission. The screening assessments will be performed as a series of short questionnaires to be self-reported on a small electronic device (such as an iPad). We anticipate it will take around 40-60 minutes to go through and we will have a researcher or staff member present to assist with any problems. Regardless of the study assessments, you would be provided with normal routine care during your stay in the hospital.

We would also collate routinely collected information from hospital systems about the clinical details of your surgical procedure and other factors (e.g. age and diabetes) that may affect your health outcomes after surgery. This information will help us understand how much the screening questionnaire results may relate to your health outcomes as opposed to other known risk factors.

After your surgery, we would follow-up your recovery until your discharge, to record if you experience any complications. At day 7 or at discharge if earlier, we would ask you to complete some of the questionnaires again to see how you are going. We will also ask you to complete a small survey about your preferences for emotional or mental health well-being support during your cardiac surgery admission. We will also ask a small number of participants to volunteer for a short interview during which you would give us more detail about your experience and preferences for emotional well-being support and stress management during the hospital admission. This interview may be conducted at time of discharge or by telephone shortly after discharge.

About a month after your operation at your out-patient clinic appointment, we would give you some of the questionnaires again. We would also ask about your health status such as if you had any complications after the operation or if you needed to get healthcare or go to the hospital for any reason after your surgery. Alternatively, we may provide you with a self-addressed stamped envelope for you to return your information to us or send you an email with a link to survey questions to enquire about your health status. We may also phone you to check in with your progress with the questionnaires. We would also like to find out about your recovery and well-being at 1 year after the operation for example, any hospital re-presentations, and public health costs from Queensland Health databases and the Queensland Births, Deaths and Marriages Registry.

Summary of participation:

Time	Questionnaires
During week before operation	Screening questionnaires<br>Well-being and Quality of Life questionnaires
1 week after operation or at discharge	Repeat some screening questionnaires<br>Well-being support preferences questionnaire
	Small number of participants for interviews on<br>well-being support preferences
1 month after operation at Outpatient clinic follow-up	Repeat some screening questionnaires<br>Repeat wellbeing and Quality of Life questionnaires
1 year after operation at Outpatient clinic follow-up	Repeat some screening questionnaires<br>Repeat well-being and Quality of Life questionnaires

That would be the end of your participation in this research project.

Bias

This research project has been designed to make sure the researchers interpret the results in a thorough, fair, and appropriate way and avoids study investigators or participants jumping to conclusions.

Additional costs

There are no additional costs associated with participating in this research project, nor will you be paid. All study assessments required as part of the research project will be provided to you free of charge.

Reimbursement

There is no reimbursement for travel, parking, meals, or other expenses associated with the research project.

Desirable to inform GP of study participation

It is desirable that your local doctor be advised of your decision to participate in this research project.

5 Other relevant information about the research project

We expect to recruit approximately 260 patients to this research project over one year only at The Prince Charles Hospital. We will be performing future separate follow-on research building on the information from this study to develop a new multi-disciplinary model of care for well-being support in cardiac surgery at The Prince Charles Hospital. The researchers are from a collaborative group between The Prince Charles Hospital, Queensland University of Technology, and the CSIRO.

6 Do I have to take part in this research project?

Participation in any research project is voluntary. If you do not wish to take part, you do not have to. If you decide to take part and later change your mind, you are free to withdraw from the project at any stage.

If you do decide to take part, you will be given this Participant Information and Consent Form to sign and you will be given a copy to keep.

Your decision whether to take part or not to take part, or to take part and then withdraw, will not affect your routine treatment, your relationship with those treating you or your relationship with The Prince Charles Hospital.

7 What are the alternatives to participation?

The extra screening assessments and questionnaires before and after surgery will only be obtained from and for people who consent to this study.

Other clinical information is routinely collected as part of the cardiac surgery care process for all patients.

You do not have to take part in this research project to receive treatment at this hospital. You can also discuss this trial with your local doctor.

8 What are the possible benefits of taking part?

The questionnaires in our study are not expected to benefit you. We hope that the results of this study will improve our knowledge of the usefulness of screening for managing cardiac surgery patient health outcomes for different patients.

If we understand who might be more at risk from surgery, precautions could be put in place, and future patients could be offered additional help to reduce the risks for worse health outcomes following their surgery.

This study may provide a basis for thinking about your own perception of well-being and stress management as part of your overall health with respect to your cardiac condition. However, there will be no direct benefit to you from your participation in this research.

9 What are the possible risks and disadvantages of taking part?

There are no expected risks or side effects to you from your participation in this research study, over and above the potential risks and side effects of your operation, other than the time related inconvenience of completing the questionnaires and possible discomfit answering some of the mental health related questions.

Participants' responses to the questionnaire will be directly collected into a research database without clinical review of individual answers. It is unlikely that the study assessments should uncover a medical condition of which the treating team were unaware, but any clinical information discovered could be referred to your surgical team. If participants wish to discuss any issues regarding answers they provide on the questionnaires, they are advised to raise such issues with their Metro North clinical care team.

Similarly, it is unlikely that participation in the research project might diagnose previously unknown conditions that may affect insurance in the future, but please consider if this might be an issue that could affect your willingness to participate.

If you become upset or distressed as a result of your participation in the research, please let the study investigators know and they will be able to refer you to your care team for appropriate support. Any support will be provided by qualified staff through the usual clinical referral process. Such support will be provided as part of the routine care services, free of charge.

Although we don't expect this to happen, if you think you have an unexpected reaction or complication from participating in this study you should contact your surgeon or research staff as soon as possible. They will help arrange the best management. Treatment of injuries or complications as a public patient in an Australian public hospital is free of charge.

10 What will happen to my test samples?

This study will not require collection of any samples or specimens.

11 What if new information arises during this research project?

It is not expected that new information will become available that would influence the study or your treatment. However, if this did happen your study investigator will tell you about it and discuss with you whether you want to continue in the research project. Your usual care will not be affected.

12 Can I have other treatments during this research project?

Participating in this trial will not affect your medical treatment; you can have all the treatments you would normally receive.

13 What if I withdraw from this research project?

If you decide to withdraw from the project, please notify a member of the research team so that we may document your withdrawal in your chart and cease study follow-up.

If you do withdraw your consent during the research project, the study investigators will not collect additional personal information from you. You may also direct us to remove your

personal information that we have already collected. We will keep evidence of your consent and later withdrawal to show we correctly followed research processes.

14 Could this research project be stopped unexpectedly?

This research project may be stopped unexpectedly for a variety of reasons. These may include reasons such as:

- The assessments being shown not to be useful
- The assessments being shown to be useful and not need further testing

15 What happens when the research project ends?

Once the study finishes, there will be no further follow-up. If the research project ends before you leave hospital, we will advise you of this.

The results of this research may be presented at scientific meetings or in publications; however, your identity will not be disclosed.

Your surgeon or member of the research team can advise you of the study results upon request.

Part 2 How is the research project being conducted?

16 What will happen to information about me?

By signing the consent form, you consent to the relevant research investigators collecting and using personal information about you for the research project. Information about you may be obtained from your health records held at this and other health services for this research. By signing the consent form, you agree to the study team accessing health records if they are relevant to your participation in this research project.

Any information obtained about this research project that can identify you will remain confidential. All the data will be stored securely according to stringent Australian and Queensland legislative and regulatory guidelines and no individual contact details or identifying information will be made available to anyone outside of the study researchers.

Before the results of this study are published, all the information that could be used to identify an individual patient will be removed. Your identifiable information will only be used for this research project and it will only be disclosed with your permission, except as required by law. It may be that further analysis of the collected information could be helpful later following the completion of this project, however in this situation, strict ethical guidelines would be followed, and all confidentiality and privacy requirements would be maintained according to Queensland and National legislation.

For those also agreeing to participate in the interview about preferences for well-being support, answers will be audio-recorded and transcribed for analysis. Some quotes from the interviews would be repeated anonymously in the reporting and write-up of the study, but we will ensure that no one can be identified from these quotes.

Your health records and any information obtained during the research project are subject to inspection (for verifying the procedures and the data) by the relevant authorities and the institution relevant to this Participant Information Sheet, The Prince Charles Hospital, or as required by law. By signing the Consent Form, you authorise release of, or access to, this confidential information to the relevant study personnel and regulatory authorities as noted above.

It is anticipated that the results of this research project will be published and/or presented in a variety of forums. In any publication and/or presentation, study information will be provided in such a way that you cannot be identified, except with your permission. Information about your participation in this research project will be recorded in your health records, including a copy of your signed consent form.

In accordance with relevant Australian and Queensland privacy and other relevant laws, you have the right to request access to your information collected and stored by the research team. You also have the right to request that any information with which you disagree be corrected. Please contact the study team member named at the end of this document if you would like to access your information.

Any information obtained for this research project and as relevant for any future follow-on studies directly related to this study that can identify you will be treated as confidential and securely stored. It will be disclosed only with your permission, or as required by law.

17 Complaints and compensation

The hospital's complaint contact person for this trial is listed over the page in section 20.

If you suffer any injuries or complications because of this research project, you should contact the study team as soon as possible and you will be assisted with arranging appropriate medical treatment. If you are eligible for Medicare, you can receive any medical treatment required to treat the injury or complication, free of charge, as a public patient in any Australian public hospital.

18 Who is organising and funding the research?

This research project is being conducted by The Prince Charles Hospital investigators, partnering with Queensland University of Technology and CSIRO.

You will not benefit financially from your involvement in this research project.

In addition, if knowledge acquired through this research leads to discoveries that are of commercial value to The Prince Charles Hospital, the study investigators or their institutions, there will be no financial benefit to you or your family from these discoveries.

No member of the research team will receive a personal financial benefit from your involvement in this research project (other than their ordinary wages).

19 Who has reviewed the research project?

All research in Australia involving humans is reviewed by an independent group of people called a Human Research Ethics Committee (HREC). The ethical aspects of this research project have been approved by the Metro North HREC.

This project will be carried out according to the *National Statement on Ethical Conduct in Human Research (2023)*. This statement has been developed to protect the interests of people who agree to participate in human research studies.

20 Further information and who to contact

The person you may need to contact will depend on the nature of your query.

If you want any further information concerning this project or if you have any medical problems which may be related to your involvement in the project, you can contact the study **Principal Investigator Susan Smith through 07 3139 4230** or any of the following people:

Clinical contact person

Name	Dr Rishendran Naidoo
Position	Consultant Cardiothoracic Surgeon
Telephone	3139 5115
Email	Rishendran.Naidoo@health.qld.gov.au

Name	Dr Usha Gurunathan
Position	Staff Specialist - Anaesthetics
Telephone	0447 189 669
Email	Usha.Gurunathan@health.qld.gov.au

Name	Dr Tricia Rolls
Position	Director Psychology, The Prince Charles Hospital
Telephone	07 3139 5935
Email	Tricia.Rolls@health.qld.gov.au

For matters relating to research at the site at which you are participating, the details of the local site complaints person are:

Complaints contact person

Position	MNHHS Research Governance Manager
Telephone	07 3647 9550
Email	MetroNorthResearch-RGO@health.qld.gov.au

If you have any complaints about any aspect of the project, the way it is being conducted or any questions about being a research participant in general, then you may contact:

Reviewing HREC approving this research and HREC Executive Officer details

Reviewing HREC name	Metro North Health HREC B
Position	Executive Officer Research
Telephone	07 3646 5280
Email	MetroNorthResearch-Ethics@health.qld.gov.au

Local HREC Office contact (Single Site -Research Governance Officer)

Position	MNHHS Research Governance Manager
Telephone	07 3647 9550
Email	MetroNorthResearch-RGO@health.qld.gov.au

Consent Form - *Adult providing own consent*

**Title**	Exploring the relationship between levels of Well-being with health outcomes and patient preferences for resources and support in the cardiac surgical setting
**Short Title**	EMBRACE: Well-Being Support in Cardiac Surgery
**NEAF Protocol Number**	80524
**Principal Investigator**s	Ms Susan Smith, Dr Esben Strodl, Dr Tricia Rolls,
**Associate Investigator(s)**	Dr Rishendran Naidoo, Dr Usha Gurunathan, Dr Marlien Varnfield, Professor David Kavanagh, Professor Jed Duff, Bo Janoschka, Dr Karen Hay
**Location**	The Prince Charles Hospital

Declaration by Participant

I have read the Participant Information Sheet or someone has read it to me in a language that I understand.

I understand the purposes, procedures and risks of the research described in the project.

I understand that there will be no *clinical* follow up from the researchers or any Metro North clinical staff regarding answers submitted in the questionnaires, unless raised by the participant.

I give permission for my doctors, other health professionals, hospitals or laboratories outside this hospital to release information to The Prince Charles Hospital concerning my disease and treatment for the purposes of this project. I understand that such information will remain confidential.

I have had an opportunity to ask questions and I am satisfied with the answers I have received.

I freely agree to participate in this research project as described and understand that I am free to withdraw at any time during the study without affecting my future health care.

I understand that I will be given a signed copy of this document to keep.

Name of Participant (please print) ______________________________

Signature ______________________ Date ______________________

Declaration by Study Doctor/Senior Researcher†

I have given a verbal explanation of the research project, its procedures and risks and I believe that the participant has understood that explanation.

Name of Study Doctor/
Senior Researcher† (please print) ______________________________

Signature ______________________ Date ______________________

† A senior member of the research team must provide the explanation of, and information concerning, the research project.

Note: All parties signing the consent section must date their own signature.

Form for Withdrawal of Participation

**Title**	Exploring the relationship between levels of Well-being with health outcomes and patient preferences for resources and support in the cardiac surgical setting
**Short Title**	EMBRACE: Well-Being Support in Cardiac Surgery
**NEAF Protocol Number**	80524
**Principal Investigators**	Ms Susan Smith, Dr Esben Strodl, Dr Tricia Rolls,
**Associate Investigator(s)**	Dr Rishendran Naidoo, Dr Usha Gurunathan, Dr Marlien Varnfield, Professor David Kavanagh, Professor Jed Duff, Bo Janoschka, Dr Karen Hay
**Location**	The Prince Charles Hospital

I wish to withdraw from participation in the above research project and understand that such withdrawal will not affect my routine treatment, my relationship with those treating me or my relationship with The Prince Charles Hospital

Name of Participant (please print) ______________________________

Signature ______________________ Date ______________________

In the event that the participant's decision to withdraw is communicated verbally, the Study Doctor/Senior Researcher will need to provide a description of the circumstances below.

Declaration by Study Doctor/Senior Researcher†

I have given a verbal explanation of the implications of withdrawal from the research project and I believe that the participant has understood that explanation.

Name of Study Doctor/
Senior Researcher† (please print) ______________________________

Signature ______________________ Date ______________________

† A senior member of the research team must provide the explanation of and information concerning withdrawal from the research project.

Note: All parties signing the consent section must date their own signature.
