# Supplementary material for: Formative research for a pre-operative psychosocial screening program for cardiac surgical patients: The EMBRACE study, a mixed methods knowledge to action protocol
Source: PLoS One. 2025 Dec 30;20(12):e0322592. doi: 10.1371/journal.pone.0322592 (PMC12752988; doi:10.1371/journal.pone.0322592)
Supplement: S1 Script — (PDF) [file pone.0322592.s005.pdf]

EMBRACE Patient Preferences: Semi-structured interview guide

Date: Interviewer: Participant UR:

Location: Teleconference: Start Time: End Time:

Participation agreement confirmation

1. As part of the EMBRACE Study we are interested in better understanding patients' preferences for emotional or mental health well-being support during cardiac surgical admission through a brief interview
2. You have already completed the survey about your preferences, and we are now inviting you to an additional short interview regarding your thoughts. Additional information is on the information sheet you have received (provide copy of EMBRACE PICF for review). Are you still interested in taking part in this part of the project? *[Await confirmation]*

   To review:

   - We do not expect any risks or discomfort associated with this research study. However, if you feel uncomfortable, you can stop the interview at any time, without giving a reason.
   - You do not have to say yes to take part; you can ask me any questions you want before or during the interview; you can also withdraw at any stage without giving a reason and without any negative consequences.
   - You do not have to answer any questions that you do not wish to.
   - You are aware that The Prince Charles Hospital Human Research Ethics committee has approved this research project; and you can contact me, or the Ethics and Research Governance officers listed on the Information sheet.
   - I will audio-record you unless you say that I can't and transcribe the conversation as part of the research and I will safely store your data electronically in encrypted, secure files within Queensland health systems.
   - I may use brief quotes of what you say during the interview in the write-up of this study, but they will remain anonymous. No-one outside the study investigators will see any identifiable information from this.
   - Are you still willing to take part?
   - Do you give your permission for me to re-contact you to clarify any information?

   [*Await confirmation]* So if you're happy with all of that, and have no more questions, let's start.

3. First, I will record your agreement to participate on audio. Do you agree to participate in the EMBRACE Patient Well-being support Preferences interview?

Participant comments on experience of emotional and/or mental health support

4. Do you think it is important for the hospital staff to recognise your emotional and/or mental health needs around your cardiac surgical admission?
   - *Do you have any general comments about your experience regarding emotional and/or mental health well-being support during your cardiac surgery stay?*
   - *Do you have any examples of how this was achieved well?*
   - *Do you have any suggestions for what could be better?*

Participant interest in types of emotional and/or mental health support

5. To better support emotional and/or mental health during cardiac surgery, what sorts of resources would you be interested in being provided to you?
   - *Do you find the printed booklets and brochures helpful?*
     - *Why/why not?*

- *Are you comfortable using electronic devices such as mobile phones and the internet?*
    - *Why/why not*
- *Do you think some electronic or on-line resources might be helpful?*
    - *Eg educational videos*
    - *On-line instructions*
    - *On-line relaxation such as breathing techniques, guided imagery, music or games?*
    - *Why/why not*
- *Have you heard of, or used on-line self-help coping and emotional supports such as Mindfulness, Positive Thinking and Cognitive Behavioural Therapy?*
    - *Do you think any of these supports might be useful to you?*
    - *Would you be interested in using any of these specifically in relation to your cardiac surgery?*
    - *Why/why not*
- *Would you prefer direct person contact such as:*
    - *From a volunteer who has experienced cardiac surgery themselves?*
    - *Or discussion with a health professional such as a psychologist, social worker, nurse, or doctor?*

Post-Discharge preferences

6. Do you think emotional and/or mental health support would be useful following discharge?
    - *Why/why not*

General follow-up questions

7. Any additional thoughts you would like to share?
8. Would you like to change or reconsider anything you have told us?
